# Population pharmacokinetic models of anti-PD-1 mAbs in patients with multiple tumor types: A systematic review

**DOI:** 10.3389/fimmu.2022.871372

**Published:** 2022-08-02

**Authors:** Jingyuan Shang, Lin Huang, Jing Huang, Xiaolei Ren, Yi Liu, Yufei Feng

**Affiliations:** ^1^ Department of Pharmacy, Peking University People’s Hospital, Beijing, China; ^2^ Faculty of Life Sciences and Biopharmaceuticals, Shenyang Pharmceutical University, Shenyang, China

**Keywords:** anti-PD-1 mAbs, immune checkpoint inhibitors, population pharmacokinetics, PPK model, systematic review

## Abstract

**Aims and background:**

A number of population pharmacokinetic (PPK) models of anti-programmed cell death-1 (PD-1) monoclonal antibodies (mAbs) in multiple tumor types have been published to characterize the influencing factors of their pharmacokinetics. This review described PPK models of anti-PD-1 mAbs that investigate the magnitude and types of covariate effects in PK parameters, provide a reference for building PPK models of other anti-PD-1 mAbs, and identify areas requiring additional research to facilitate the application of PPK models.

**Methods:**

A systematic search for analyses of PPK models of eleven anti-PD-1 mAbs on the market that were carried out in humans was conducted using PubMed, Embase, and the Cochrane Library. The search covered the period from the inception of the databases to April 2022.

**Results:**

Currently, there are fourteen analyses on PPK models of anti-PD-1 mAbs summarized in this review, including seven models that refer to nivolumab, four referring to pembrolizumab, one referring to cemiplimab, one referring to camrelizumab, and one referred to dostarlimab. Most analyses described the pharmacokinetics of anti-PD-1 mAbs with a two-compartment model with time-varying clearance (CL) and a sigmoidal maximum effect. The estimated CL and volume of distribution in the central (V_C_) ranged from 0.179 to 0.290 L/day and 2.98 to 4.46 L, respectively. The median (range) of interindividual variability (IIV) for CL and V_C_ was 30.9% (8.7%–50.8%) and 29.0% (4.32%–40.7%), respectively. The commonly identified significant covariates were body weight (BW) on CL and V_C_, and albumin (ALB), tumor type, sex, and performance status (PS) on CL. Other less assessed significant covariates included lactate dehydrogenase (LDH), immunoglobulin G (IgG), ipilimumab coadministration (IPICO) on CL, and body mass index (BMI), malignant pleural mesothelioma (MESO) on V_C_.

**Conclusion:**

This review provides detailed information about the characteristics of PPK models of anti-PD-1 mAbs, the effects of covariates on PK parameters, and the current status of the application of the models. ALB, BW, specific tumor type, sex, and PS should be considered for the future development of the PPK model of anti-PD-1 mAbs. Other potential covariates that were assessed less frequently but still have significance (e.g., LDH, IgG, and IPICO) should not be ignored. Thus, further research and thorough investigation are needed to assess new or potential covariates, which will pave the way for personalized anti-PD-1 mAbs therapy.

## Background

In recent years, immune checkpoint inhibitors (ICIs) have markedly transformed the treatment of multiple cancers. Immune checkpoints consist of a group of regulatory surface proteins that are entrenched in the immune system and are crucial for preventing autoimmune responses. Current immune checkpoints targeted by ICI include cytotoxic T-lymphocyte protein 4 (CTLA-4), PD-1, and programmed cell death-ligand 1 (PD-L1) (1). To date, blocking the interactions between PD-1 and PD-L1 is the most prominent and effective strategy.

PD-1, also known as CD279, is an inhibitory immune checkpoint receptor expressed primarily on T cells and is associated with reduced T-cell activity and exhaustion (2, 3). Pembrolizumab (Keytruda) and nivolumab (Opdivo) were the first two anti-PD-1 mAbs that received approval by the FDA in September and December 2014, respectively (4). In the upcoming years, several novel mAbs against PD-1, cemiplimab (5), camrelizumab (6), tislelizumab (7), toripalimab (8), sintilimab (9), prolgolimab (10), dostarlimab (11), penpulimab (12), and zimberelimab (13) received approval for marketing in different countries consecutively. Anti-PD-1 mAbs have shown long-lasting objective responses in different tumor types, such as non-small cell lung cancer (NSCLC), renal cell carcinoma (RCC), gastric carcinoma (GC), classical Hodgkin’s lymphoma (cHL), melanoma (MEL), endometrial carcinoma (EC), etc. (14).

Anti-PD-1 mAbs with enormous molecular weights have more complex pharmacokinetic features than standard small molecules (15). They have a limited volume of distribution and are thought to be largely confined to the vascular and interstitial spaces. They are primarily eliminated *via* three mechanisms: a non-specific clearance with pinocytosis by vascular endothelial cells; a specific target-mediated drug disposition caused by the specific Fab region of the antibody-antigen-mediated endocytosis; and a non-specific receptor-mediated endocytosis through the Fc domain of the antibody binding with Fc gamma receptor (FcγR)-expressing cells (16). Although currently marketed mAbs are mostly of the IgG_1_ subclass, approved anti-PD-1 mAbs are IgG_4_. IgG_4_ mAbs have a weaker affinity of the Fc domain for FcγR; thus anti-PD-1 mAbs can preserve the activated bound T cells from antibody-dependent cellular cytotoxicity (ADCC) and antibody-dependent cell phagocytosis (ADCP) (17). In contrast with these clearance mechanisms, the neonatal Fc receptor (FcRn) binds to the Fc fragment of mAbs in a pH-dependent manner to prevent mAbs from rapid intracellular catabolism, which explains the relatively long half-life of anti-PD-1 mAbs (16, 18).

Despite remarkable success in a subset of patients, a large IIV has been observed in the efficacy of anti-PD-1 mAbs. Liu et al. found that the CL of nivolumab decreased with improving disease dynamics over time, and complete responders had the largest reduction in CL, while patients with disease progression showed the smallest reduction or even increased CL (19). Studies of other anti-PD-1 mAbs have also shown that the efficacy of treatment outcomes correlated negatively with CL (20–23). In such cases, employing the PPK model can describe the typical pharmacokinetic parameters of the target population, attempt to determine the measurable factors affecting the PK of anti-PD-1 mAbs, and identify the magnitude of these effects (24).

Currently, several PPK models for anti-PD-1 mAbs have been developed in patients with various tumor types. However, no research summarizing or evaluating PPK modeling of anti-PD-1 mAbs has been published yet. In consideration of the complex PK process of anti-PD-1 mAbs, it has become challenging to select an appropriate basic PK model and identify the significant covariates affecting the PK of anti-PD-1 mAbs. As a result, it is vital to make better use of the strategy of existing models and additional considerations for developing new models. To our knowledge, this review is the first study to systematically investigate the PPK models of anti-PD-1 mAbs that are currently accessible. Our research presented an overview of these published PPK studies, investigated clinical determinants influencing the PK of anti-PD-1 mAbs and identified areas requiring additional research.

## Methods

### Search strategy

An electronic literature search was performed using PubMed, Embase, and the Cochrane Library to identify PPK analyses of anti-PD-1 mAbs for the entire time period from inception to April 2022. The pertinent PPK analyses on anti-PD-1 mAbs were identified using the following search terms: programmed death-1, nivolumab, pembrolizumab, cemiplimab, camrelizumab, tislelizumab, toripalimab, sintilimab, prolgolimab, dostarlimab, penpulimab, zimberelimab, population pharmacokinetic, nonlinear mixed effect, NONMEM, etc. Further, a thorough inspection of all the pertinent lists of references was conducted to identify any additional relevant materials.

### Inclusion criteria and exclusion criteria

Analyses were included in this systematic review if they were (1) conducted on humans, (2) based on the use of anti-PD-1 mAbs as the treatment, (3) providing PPK analyses of anti-PD-1 mAbs, and (4) employing at least one type of PPK analysis. However, the publication was excluded if (1) the details of methodology or pharmacokinetics were insufficient, (2) it was a review or methodology study, and (3) it excluded the model development process.

### Data extraction

A reviewer-extracted information related to the study design, population baseline characteristics, and PPK analyses, which were essential for interpreting the results. The second reviewer independently checked the data extraction to minimize errors. A standard data collection form was used to extract the following information from each eligible study: study design (e.g., number of patients and samples, data source, dosage regimen, methods of concentrations determination), patient population characteristics (e.g., sex, age, body weight, race, and tumor type), and PPK analyses (e.g., software, pharmacokinetic model, CL type, tested covariates, methods of screening covariates, residual error type, covariates of the final model and their relationship with pharmacokinetic parameters, and model evaluation).

### Quality analysis

The methodological quality of each included analysis was assessed by the National Institutes of Health (NIH) study quality assessment tool for case series studies in this review. This tool was developed by methodologists and the NIH based on quality assessment methods and concepts and can be used for nonrandomized studies and case series, which are commonly applied in systematic reviews that include observational studies (25).

### Covariate effects

The effects of significant covariates on CL and V_C_ in each study were summarized using a forest plot, which was implemented using R software (version 4.2.0). We scaled continuous covariates, such as BW, estimated glomerular filtration rate (eGFR), and ALB, to the same range for comparison. The range was set using the 5th to 95th percentile from the analysis with the largest sample size. If the covariate was identified in only one study, the minimum and maximum values were used. The reference values were the data provided in the PPK models. If the reference value was not mentioned, the median value was used. For categorical covariates, such as tumor type, 0 and 1 were used. Since only the final model was available for most published PPK analyses, the analyses in this manuscript used the estimates of covariate effects from the final PPK models.

According to the determined range of covariates and the parameter estimates (95% confidence interval [CI]) provided, the minimum and maximum CL and V_C_ values (95% CI) were calculated. The effect of the identified covariate on CL and V_C_ in each study was displayed by the following equation (Eq. 1) (26):


(1) $${\text{Covariate}}_{\text{j}}\;{\text{effect in study}}_{\text{i}}\text{ = }\frac{\text{The range of calculated CL or Vc}}{{\text{The CL or Vc reference in study}}_{\text{i}}}\times 100\%$$

where study_i_ is the the ith study and covariate_j_ is the jth identified covariates in study_i_.

The assessment of the magnitude of the covariate effect on CL or V_C_ was based on an 80%–120% boundary, which was used as a screening criterion for potential clinical significance. The covariate effect within 80%–120% was not expected to have clinical significance (27).

## Results

### Study characteristics and quality assessment

**Figure 1:**
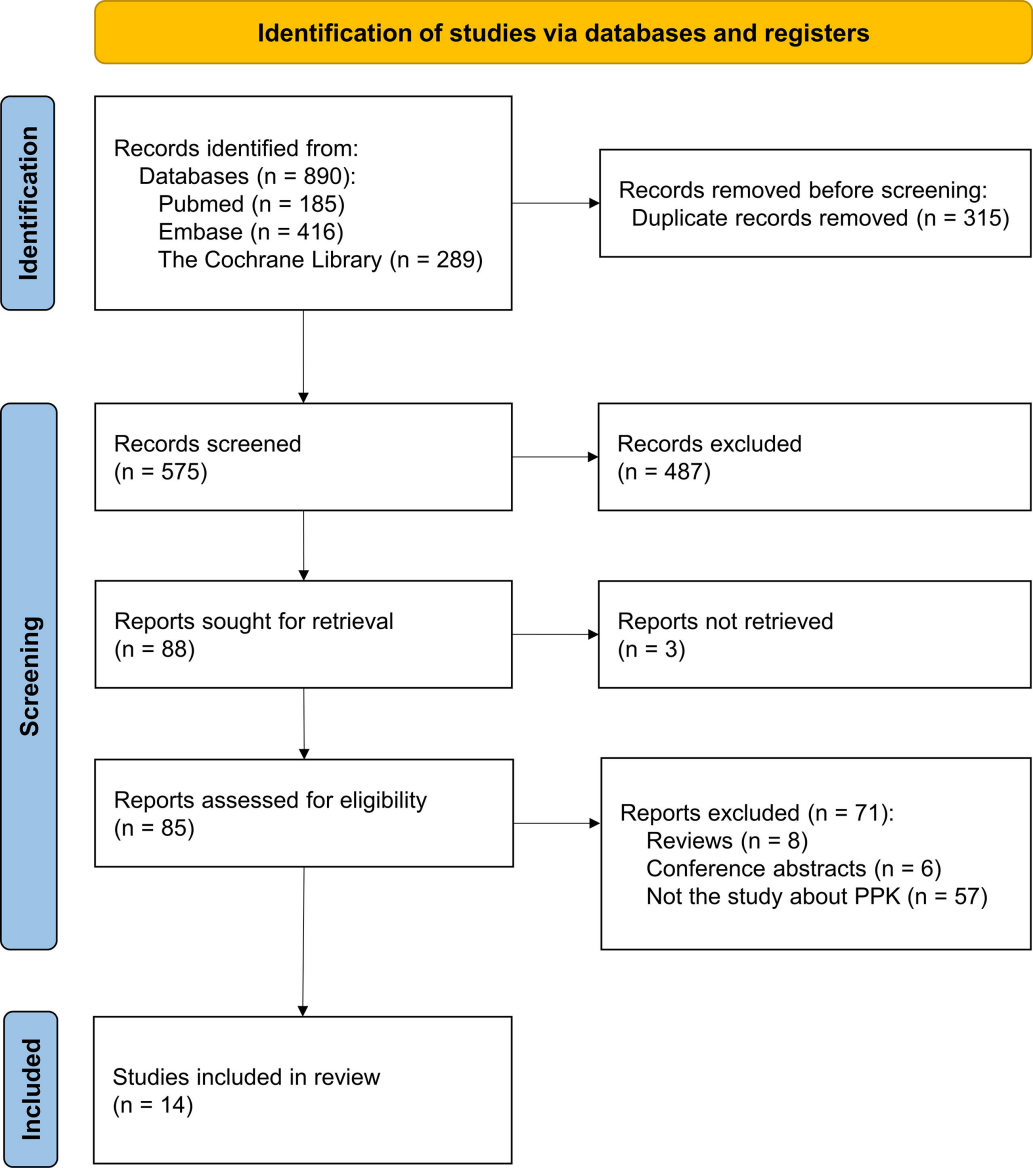
The PRISMA flow diagram.

Among the 279 documents retrieved, a total of fourteen analyses were included in the review for further analysis (**Figure 1**). Using the NIH study quality assessment tool, fourteen analyses had a good quality rating. **Table 1** summarizes the characteristics of the included analyses, among which seven referred to nivolumab (21, 22, 28–32), four referred to pembrolizumab (20, 23, 33, 34), one referred to cemiplimab (35), one referred to camrelizumab (36), and one referred to dostarlimab (37). The median (range) number of patients used to develop the PPK model was 1,137 (122–6,848) in fourteen analyses, with eight analyses (nivolumab 6/7, pembrolizumab 2/4) (21, 28–34) involving more than 1,000 subjects. The data sources of twelve analyses were gathered from different clinical trials (nivolumab 6/7, pembrolizumab 3/4, cemiplimab 1/1, camrelizumab 1/1, and dostarlimab 1/1) (20, 21, 28–37), and two analyses were prospectively collected from a real-life patient cohort (nivolumab 1/7, pembrolizumab 1/4) (22, 23). All fourteen analyses used internal evaluation to assess the model, of which nine analyses (nivolumab 4/7, pembrolizumab 3/4, cemiplimab 1/1, and camrelizumab 1/1) used three or more methods of internal evaluation (21–23, 28, 32–36). However, none of the analyses have been externally validated.

**Table 1:** Characteristics of the included analyses [n = 14].

Characteristics	No. of analyses [n (%)]
Drugs studied	
Nivolumab	7 (50.0%)
Pembrolizumab	4 (28.6%)
Cemiplimab	1 (7.1%)
Camrelizumab	1 (7.1%)
Dostarlimab	1 (7.1%)
Number of patients	median 1,137 (range 122–6,468)
Number of samples	median 8,585 (range 600–32,835)
Data source	
Clinical trials	12 (85.7%)
Real-world studies	2 (14.3%)
Methods of concentrations determination	
Ligand-binding ELISA or ELC	3 (21.4%)
ELISA	4 (28.6%)
ELC-based immunoassay	1 (7.1%)
Unspecified	6 (42.9%)
Best structural pharmacokinetic model	
Two-compartment	13 (92.9%)
One-compartment	1 (7.1%)
CL type	
Time-varying CL	11 (78.6%)
Time-stationary CL	3 (21.4%)
Residual error models	
Proportional	8 (57.1%)
Log- transformed additive	2 (14.3%)
Combined proportional and additive	3 (21.4%)
Unspecified	1 (7.1%)
Numbers of model evaluation methods used (GOF, VPC, bootstrap analysis, posterior predictive check)	
≥3 methods	9 (64.3%)
2 methods	5 (35.7%)

ELISA, enzyme-linked immunosorbent assay; ELC, electrochemiluminescence; CL, clearance; GOF, goodness-of-fit; VPC, visual predictive checks.

The population of included analyses contained a large number of patients of different races (White, African American, Asian, Chinese, etc.) and multiple tumor types (NSCLC, MEL, RCC, HCC, CRC, NPC, EC, etc.). The specific information about the gender, age, and BW of the patients is summarized in **Table 2**. The dosage regimen of anti-PD-1 mAbs was intravenous infusion of either a weight-based (nivolumab 0.1–20.0 mg/kg every 2 weeks or 3 weeks, pembrolizumab 1–10 mg/kg every 2 weeks or 3 weeks, cemiplimab 1, 3, 10 mg/kg every 2 weeks or 3 mg/kg every 3 weeks, camrelizumab 1, 3, 10 mg/kg every 2 weeks), or a fixed regimen (nivolumab 240 mg every 2 weeks, pembrolizumab 200 mg every 2 weeks, cemiplimab 200 mg every 2 weeks or 350 mg every 3 weeks, camrelizumab 60, 200, or 400 mg every 2 weeks, dostarlimab 500 mg every 3 weeks four cycles followed by 1,000 mg every 6 weeks). The serum concentrations of anti-PD-1 mAbs were quantified using an enzyme-linked immunosorbent assay (ELISA) (22, 23, 29–31, 36, 37), electrochemiluminescence (ELC) (29–31), or an ELC-based immunoassay method (33).

**Table 2:** Studies design and characteristics of the population.

MAbs	Analyses	Patients (n)	Samples(n)	Male (%)	Female (%)	Age(year) Mean ± SD Median [range]	Body weight (kg)Mean ± SD Median [range]	Race (%)	Cancer (%)	Drug dose	Data source	Sample assay
Nivolumab	Bajaj et al. (28)	1,895	12,292	1,264 (66.7%)	631 (33.3%)	61.1 ± 11.1	79.1 ± 19.3	White (88.92%) African American/Black(2.8%) Asian (6.44%) Other (1.74%)	MEL (29.82%) NSCLC (34.78%) RCC (31.93%) Other (3.48%)	Nivolumab 0.3–10.0 mg/kg Q2W or Q3W IV infusion	11 clinical trails (MDX1106-01, ONO-4538-01, MDX1106-03, CA209010, CA209063, ONO-4538-02, CA209017, CA209037, CA209025, CA209057, CA209066)	NA
Nivolumab	Hamuro et al. (21)	1,773	11,644	1,088 (61.4%)	685 (38.6%)	NA	77 [34–160]	White (90.4%) African American (2.8%) Asian (5.5%) Other/missing (1.3%)	adjMEL (25.67%) MEL (31.87%) NSCLC 2L+ (34.78%) Other (6.37%)	Nivolumab 0.1–20 mg/kg Q2W IV infusion	10 clinical studies (CA209-001, CA209-003, CA209-005, CA209-063, CA209- 051, CA209- 017, CA209- 057, CA209- 037, CA209- 066, CA209- 238)	NA
Nivolumab	Hurkmans et al. (22)	221	1,715	138 (62.4%)	83 (37.6%)	65 [59-71]	78 [70–88]	Caucasian (88.2%) Other (2.3%) Unknown (9.5%)	NSCLC (71.5%) RCC (6.3%) MEL (21.7%) MESO (0.5%)	Nivolumab 3 mg/kg Q2W IV infusion	Real-world population (Dutch Trial Register number NTR7015/ NL6828)	ELISA
Nivolumab	Osawa et al. (29)	1,302	8,585	847 (65.03%)	455 (34.95%)	NA	80 [45–105]	Asian (30.65%) African American (3.92%) White/Other (63.98%)	GC/GEJC (29.72%) NSCLC 2L+ (49.69%) Other (20.58%)	Nivolumab 0.1–10 mg/kg (single dose or Q2W) IV infusion	nine clinical studies (CA209001, CA209003, ONO-4538-01, CheckMate 032, CA209063, ONO-453802, CheckMate 017, CheckMate 057, ATTRACTION-2)	Two different ligand-binding ELISAs and an ECL assay
Nivolumab	Wang, et al. (30)	1,074	NA	659 (61.36%)	415 (38.64%)	61 [27–78]	73 [49–109]	NA	cHL (17.97%) NSCLC (61.36%) Other (20.67%)	Nivolumab 0.1–20 mg/kg Q2W or Q3W IV infusion	nine clinical studies (MDX-1106-01, ONO-4538-01, MDX-110603, CA209-039, ONO-4538-02, CA209-063, CA209-205, CA209-017, CA209-057)	A ligand-binding ELISA or an ECL assay
Nivolumab	Zhang et al. (31)	1,200	6,954	812 (67.67%)	388 (32.33%)	NA	73.5 ± 17.3	Chinese (26.17%) Non-Chinese (1.75%) Non-Asian (72.08%)	NSCLC (80.5%) NPC (1.92%) Other (17.58%)	Nivolumab 0.1–10 mg/kg Q2W; 240 mg Q2W IV infusion	two Chinese (CheckMate 077 and CheckMate 078) and five global studies (MDX1106-01, CA209-003, CheckMate 017, CheckMate 057 and CheckMate 063)	A ligand-binding ELISA or an ECL assay
Nivolumab	Zhang et al. (32)	6,468	32,835	4,214 (65.15%)	2,254 (34.85%)	NA	77.6 ± 18.8	White/other (85.58%) African American (2.32%) Asian (10.33%)	NSCLC (38.25%) MEL (26.93%) RCC (19.25%) SCLC (6.03%) HCC (5.89%) CRC (3.65%)	Nivolumab monotherapy or nivolumab in combination with ipilimumab or chemotherapy	25 clinical studies (CA209-009, CA209-010, CA209-012, CA209-016, CA209-017, CA209-025, CA209-026, CA209-032, CA209-037, CA209-057, CA209-063, CA209-066, CA209-067, CA209-069, CA209-214, CA209-227, MDX1106-01, MDX1106-03, MDX1106-04, ONO-4538-01, ONO-4538-02, CA209-511, CA209-568, CA209-040, CA209-142)	NA
Pembrolizumab	Ahamadi et al. (33)	2,195	12,171	1293 (59.1%)	865 (40.9%)	62 [15–94]	NA	NA	MEL (73.7%) NSCLC (25.3%) Other (1.01%)	Pembrolizumab 1–10 mg/kg Q2W or Q3W IV infusion	three clinical trials (KEYNOTE-001, KEYNOTE-002, KEYNOTE-006)	An ELC-based immunoassay method
Pembrolizumab	Li et al. (34)	2841	19,042	1,691 (59.5%)	1,150 (40.5%)	61.0 ± 12.5	77.2 ± 18.9	White (88.6%) Black (1.7%) Asian (8.1%) Other/missing (1.6%)	MEL (56.7%) NSCLC (42.5%) Missing (0.8%)	Pembrolizumab 1–10 mg/kg Q2W or Q3W IV infusion	four clinical trials (KEYNOTE-001, KEYNOTE-002, KEYNOTE-006, KEYNOTE-010)	NA
Pembrolizumab	Li et al. (20)	644	3,909	391 (60.7%)	253 (39.3%)	Mean 62.1	Mean 71.3	White (71.1%) Black (3.3%) Asian (21.6%) Other (4.1%)	NSCLC (100%)	Pembrolizumab 2 mg/kg Q3W; 10 mg/kg Q3W IV infusion	one clinical trial (KEYNOTE-010)	NA
Pembrolizumab	Hurkmans et al. (23)	122	600	80 (65.6%)	42 (34.4%)	69 [57–74]	80 [68-90]	Caucasian (91.0%) Other (1.6%) Unknown (6.6%)	NSCLC (34.4%) MEL (41.8%) UCC (12.3%) MESO (10.7%) SCLC (0.8%)	Pembrolizumab 2 mg/kg Q3W or 200 mg Q3W IV infusion	Real-world population (Dutch Trial Registry Number NL6828)	ELISA
Cemiplimab	Yang et al. (35)	548	11,178	331 (60.4%)	217 (39.6%)	65 [27–96]	76 [31–172]	White (90.9%) Black (3.6%) Asian (1.6%) Other (3.8%)	CSCC (32.48%) Other (67.52%)	Cemiplimab 1, 3, or 10 mg/kg Q2W, or 3 mg/kg Q3W, or 200 mg Q2W, or 350 mg Q3W IV infusion	two clinical studies (NCT02383212, NCT02760498)	NA
Camrelizumab	Wang et al. (36)	133	3,298	88 (66.2%)	45 (33.8%)	50 [21–69]	61 [37–91]	Han (96.2%) Other (3.8%)	NPC (25.6%) LC (15.8%) MEL (27.1%) ESCA (10.1%) GC (3.8%) cHL (9.0%) Other (8.2%)	Camrelizumab 1 mg/kg, 3 mg/kg, 10 mg/kg Q2W or 60 mg, 200 mg, 400 mg Q2W IV infusion	four clinical trials from China (SHR-1210-101, SHR-1210-102, SHR-1210-103, SHR-1210-II-204)	ELISA
Dostarlimab	Melhem et al. (37)	546	4,783	124 (22.7%)	422 (77.3%)	62.5 ± 11.0	74.4 ± 20.0	White (75.1%) Black/African American (3.5%) Asian (2.4%) Other (1.1%) Unknown (17.2%)	MMRp/MSS EC (29.3%) Non-EC MSI-H and POLE-Mut (28.8%) dMMR/MSI-H EC (23.4%) NSCLC (12.3%) Missing (6.2%)	Dostarlimab 500 mg Q3W × four cycles followed by 1,000 mg Q6W	Phase 1 GARNET (NCT02715284) trial	ELISA

MAbs, monoclonal antibodies; SD, standard deviation; MEL, melanoma; NSCLC, non-small cell lung cancer; RCC, renal cell carcinoma; Q2/3W, once every 2/3 weeks; IV, intravenous injection; NA, not available; adjMEL, adjuvant therapy for patients with melanoma whose tumors were removed by surgical resection; 2L+, second- line therapy or greater; SCLC, small cell lung cancer; HCC, hepatocellular carcinoma; CRC, colorectal cancer; NPC, nasopharyngeal carcinoma; ELISA, enzyme-linked immunosorbent assay; ELC, electrochemiluminescence; cHL, classical Hodgkin’s lymphoma; GC/GEJC, gastric cancer or gastro-esophageal junction cancer; MESO, malignant pleural mesothelioma; UCC, urothelial cell cancer; CSCC, cutaneous squamous cell carcinoma; LC, lung cancer; ESCA, esophageal cancer; MMRp, mismatch repair proficient; MSS, microsatellite stable; EC, endometrial cancer; MSI-H, microsatellite instability-high; POLE-Mut polymerase ε mutated.

### Population pharmacokinetic analyses

Information about the selected structural models, CL types, estimates of typical pharmacokinetic parameters, pharmacokinetic parameters and covariate relationships, IIV, residual error, and PPK model evaluation for each analysis are summarized in **Table 3**. All the PPK models were developed using NONMEM, an industry-standard software program.

**Table 3:** Modeling strategies and PK parameters of published PPK analyses of anti-PD-1 mAbs.

MAbs	Analyses	PK model	CL type	CL(L/Day)	Q (L/Day)	V_C_(L)	V_P_(L)	Pharmacokinetic parameters and covariates relationships		IIV	Residual error	PPK model evaluation
								CL	V_C_			
Nivolumab	Bajaj et al. (28)	Two-compartment, zero-order infusion, first-order elimination	Time-varying CL with a sigmoidal maximum effect (E_max_) model	0.225	0.77	3.63	2.78	$\begin{array}{l} {\text{CL}}_{\text{t},\text{i}}\\ =0.225\times {\left(\text{BBWT}/80\right)}^{0.566}\\ \times \;{\left(\text{eGFR}/90\right)}^{0.186}\times {\left({\text{e}}^{0.172}\right)}^{\text{PS}}\\ \times {\left({\text{e}}^{0.165}\right)}^{\text{SEX}}\times {\left({\text{e}}^{-0.125}\right)}^{\text{RAAS}}\\ \times \text{ exp}\left(\frac{{\text{E}}_{\text{maxi}}\times T^{\text{γ}}}{{\text{T}}_{50\text{i}}^{\gamma }+T^{\text{γ}}}\right) \end{array}$	V_C_=3.63 × (BBWT/80)^0.597^ × (e^0.152^)^SEX^	CL: 35% V_C_: 35.1%	Proportional residual error model (21.5%)	GOF plots VPC bootstrap
Nivolumab	Hamuro et al. (21)	Two-compartment, zero-order infusion, first-order elimination	AdjMEL: stationary CL other tumor types: time- varying CL with a sigmoidal- E_max_ model	0.259	0.689	4.01	2.78	$\begin{array}{l} {\text{CL}}_{\text{t},\text{i}}\\ =0.259\;\times \;{\left(\text{BBWT}/80\right)}^{0.6\;}\\ \times \left(\text{BGFR}/90\right){\;}^{0.12}\times {\left({\text{e}}^{-0.14}\right)}^{\text{SEX }}\\ \times \;{\left({\text{e}}^{0.170}\right)}^{\text{PS}}\;\times {\left({\text{e}}^{0.0147}\right)}^{\text{RAAA}}\;\\ \times \;{\left({\text{e}}^{-0.0731}\right)}^{\text{RAAS}}\;\times \;{\left({\text{e}}^{0.0268}\right)}^{\text{OTHER}}\\ \times \;{\left({\text{e}}^{-0.514}\right)}^{\text{AdjMEL}}\;\times \;{\left({\text{e}}^{-0.0361}\right)}^{\text{NSCLC}}\;\\ \times \text{ exp}\left(\frac{{\text{E}}_{\text{maxi}}\times T^{\text{γ}}}{{\text{T}}_{50\text{i}}^{\gamma }+T^{\text{γ}}}\right) \end{array}$	V_C_=4.01 ×(BBWT/80)^0.55^ ×(e^−0.153^)^SEX^	CL: 31.1% V_C_: 37.3%	Proportional residual error model (100%)	GOF plots VPC bootstrap
Nivolumab	Hurkmans et al. (22)	Two-compartment	Time-stationary CL	0.211	0.48	3.46	3.46	CL=Female gender (−0.17)+BSA(0.97) + ALB(−1.34)	–	CL: 30.7%	Proportional residual error model (31.8%)	GOF plots VPC bootstrap
Nivolumab	Osawa et al. (29)	Two-compartment, zero-order infusion and first-order elimination	Time-varying CL with a sigmoidal maximum effect (E_max_) model	0.264	0.624	4.46	2.52	CL=BBWT(0.498)+GFR(0.151)+ SEX(−0.134)+PS(0.117)+ OTH(0.128)+GC(0.31)+ RAAA(−0.049)+RAAS(−0.201)+ BALB(−0.869)+BLDH(0.379)+ BTSIZE(0.089)+CASG(−0.193)+ CASG_MIS(−0.112)	V_C_= BBWT(0.428) + SEX(−0.189)	CL: 30.7% V_C_: 32.9%	Proportional residual error model (21.9%)	VPC bootstrap
Nivolumab	Wang et al. (30)	Two-compartment, zero-order infusion, first-order elimination	Time-varying CL with a sigmoidal maximum effect (E_max_) model	0.259	0.746	4.13	2.50	$\begin{array}{l} {\text{CL}}_{\text{t},\text{i}}\\ =0.259\;\times \;{\left(\text{BBWT}/80\right)}^{0.754}\\ \times \;{\left(\text{BGFR}/80\right)}^{0.163}\;\\ \times \;{\left(\text{BALB}/4\right)}^{-0.711\;}\times \;{\left({\text{e}}^{0.0802}\right)}^{\text{PS}}\\ \times \;{\left(\text{AGE}/61\right)}^{0.36}\\ \times \text{ exp}\left(\frac{{\text{E}}_{\text{maxi}}\times T^{\text{γ}}}{{\text{T}}_{50\text{i}}^{\text{γ}}+T^{\text{γ}}}\right) \end{array}$	V_C_=4.13× (BBWT/80) ^0.615^× (e^0.102^)^SEX^ ×(e^−0.16^)^SQ|NSQ^	CL: 10.5% V_C_: 25.6%	Proportional residual error model (2.01%)	GOF plots VPC
Nivolumab	Zhang et al. (31)	Two-compartment, zero-order infusion, first-order elimination	Time-varying CL with a sigmoidal maximum effect (E_max_) model	0.278	0.703	4.19	2.64	$\begin{array}{l} {\text{CL}}_{\text{t},\text{i}}\\ =0.278\;\times \;\left(\text{BBWT}/80\right){\;}^{0.529}\\ \times \;{\left(\text{eGFR}/90\right)}^{0.132}\;\times \;{\left({\text{e}}^{-0.182}\right)}^{\text{SEX}}\;\\ \times \;\left({\text{e}}^{0.138}\right){\;}^{\text{PS}}\times \;{\left({\text{e}}^{-0.00409}\right)}^{\text{RAAA}}\\ \times \;\left({\text{e}}^{-0.0891}\right){\;}^{\text{RAAS}}\times \;{\left({\text{e}}^{0.0889}\right)}^{\text{NPC }}\\ \times \;\left({\text{e}}^{0.0718}\right){\;}^{\text{OTHER}}\\ \times \text{ exp}\left(\frac{{\text{E}}_{\text{maxi}}\times T^{\text{γ}}}{{\text{T}}_{50\text{i}}^{\text{γ}}+T^{\text{γ}}}\right) \end{array}$	V_C_= 4.19 × (BBWT/80)^0.74^ × (e^−0.132^)^SEX^	CL: 34.5% V_C_: 31.8%	Proportional residual error model (22.4%)	VPC bootstrap
Nivolumab	Zhang et al. (32)	Two-compartment, zero-order infusion	Time-varying CL with a sigmoidal maximum effect (E_max_) model	0.259	0.838	4.27	2.70	$\begin{array}{l} {\text{CL}}_{\text{t},\text{i}}\\ =0.259\;\times \;{\left(\text{BBWT}/80\right)}^{0.53\;}\\ \times \;{\left(\text{eGFR}/90\right)}^{0.202}\;\times \left({\text{e}}^{0.227}\right){\;}^{\text{IPI}3\text{Q}3\text{W}}\\ \times \;{\left({\text{e}}^{0.159}\right)}^{\text{IPI}1\text{Q}6\text{W}}\times \;{\left({\text{e}}^{-0.104}\right)}^{\text{CHEMO}}\\ \times \;\left({\text{e}}^{-0.181}\right){\;}^{\text{SEX}}\times \;{\left({\text{e}}^{0.181}\right)}^{\text{PS}}\;\\ \times \;{\left({\text{e}}^{0.0374}\right)}^{\text{RAAA}}\;\times {\left({\text{e}}^{-0.0354}\right)}^{\text{RAAS}}\\ \times \;{\text{e}}^{\text{ηCLi}}\;\times \text{ exp}\left(\frac{{\text{E}}_{\text{maxi}}\times T^{\text{γ}}}{{\text{T}}_{50\text{i}}^{\text{γ}}+T^{\text{γ}}}\right) \end{array}$	V_C_=4.27 × (BBWT/80)^0.534^× (e^−0.161^)^SEX^×e^*η*V_Ci_^	CL: 39.6% V_C_: 39%	Proportional residual error model (24.5%)	GOF plots VPC bootstrap
Pembrolizumab	Ahamadi et al. (33)	Two-compartment, linear CL	Time-stationary CL	0.22	0.795	3.48	4.06	CL=0.22 ×(BBWT/76.8)^0.578^× (ALB/39.6)^−0.907^ × (BSLD/89.6)^00872^× (eGFR/88.47) ^0.135^× [ (1 – 0.152) if female ]× [ (1+ 0.145) if NSCLC ]× [ (1+0.0739) if baseline ECOG numeric=1 ]× [ (1+0.140) if IPI=prior treatment ]	V_C_=3.48 × (BWT/76.8) ^0.492^×(ALB/39.6) ^−0.208^× [ (1−0.134) female ]× [ (1+0.0736) IPI =prior treatment ) ]	CL or Q: 38% V_C_ or V_P_: 21%	Log-transformed additive residual error model (27.2%)	GOF plots VPC bootstrap a posterior predictive check approach
Pembrolizumab	Li et al. (34)	Two-compartment	Time-varying CL (a time dependent PK component was implemented on the CL)	0.249	0.889	3.47	2.96	CL=ALB (−0.941)+BIL(−0.0497)+ CANC(0.0544)+eGFR(0.116)+ PS (0.0636)+ SEX(−0.162)+ BSLD (0.111 )	V_C_=(ALB(−0.226)+ SEX(−0.128)	CL or Q: 30.7% V_C_ or V_P_: 19.6%	Log-transformed additive residual error model (25.1%)	GOF plots VPC bootstrap
Pembrolizumab	Li et al. (20)	Two-compartment	Time-varying CL (4 time-varying covariates: R_SLD_ R_LC_ R_ALB_ R_LDH_)	0.238	0.807	3.34	3.62	CL_t,i_ =CL_baseline_ × F_CL_ × TMPK +CL_baseline_ × (1− F_CL_) TMPK=[ R_SLD_(t) ]^TSE^ × [ R_LC_(t) ]^LCE^ × [ R_ALB_(t) ]^ASE^ × [ R_LDH_(t) ]^LDE^ CL_baseline_=0.238× (WT/75)^P^ × CoCoν × CaCoν × e^*η*i^	V_C_= ALB(−0.268) + SEX(−0.136)	CL or Q: 26.1% V_C_ or V_P_: 17.2%	NA	GOF plots bootstrap
Pembrolizumab	Hurkmans et al. (23)	One-compartment	Time-stationary CL	0.257	–	6.80	–	CL=BSA(1.46) + ALB(−1.43) + UCC(1.29)	V=MESO(0.58)+ LDH(0.34)	CL: 31% V: 29%	Proportional residual error model (17%)	GOF plots VPC bootstrap
Cemiplimab	Yang et al. (35)	Two-compartment, zero-order infusion, first-order elimination	Time-varying CL with a sigmoidal maximum effect (E_max_) model	0.290	0.638	3.32	1.65	$\begin{array}{l} {\text{CL}}_{\text{t},\text{ i}}\;\\ =0.29\;\times \;\left(\text{BBWT}/\text{BBWTREF}\right){\;}^{0.447}\\ \times \;\left(\text{BALB}/\text{BALBREF}\right){\;}^{-0.926}\;\\ \times \;{\left(\text{BIgG}/\text{BIgGREF}\right)}^{0.184}\\ \times \;{\left(\text{BALT}/\text{ BALTREF}\right)}^{\;-0.0795}\;\\ \times \text{ exp}\left(\frac{{\text{Emax}}_{\text{i}}*T^{\text{y}}}{\text{T}50_{i}^{\text{y}}+T^{\text{y}}}\right)\;\times \text{ exp}\left({\text{n}}_{\text{i}}\right) \end{array}$	V_C_=3.32 × (BWT/ BWTREF) ^0.97^× (BBMI/ BBMIREF)^ −0.56^ × exp(n_i_)	CL, Q: 8.70% V_ss_: 4.32%	A combined proportional (18.8%) and additive (1.48 mg/L) error model	GOF plots VPC bootstrap
Camrelizumab	Wang et al. (36)	Two-compartment, parallel linear and nonlinear clearance	Parallel linear and nonlinear CL CL_linear_=K_linear_×V_1_ CL_nonlinear_=V_m_/(K_m_+C_1_)	0.231	0.414	3.07	2.90	CL_liner_ =0.231 × (ALB/ 44)^−1.98^ × e^*η*CL^	V_C_=3.07 × e^*η*Vc^	CL_line_: 50.8% V_C_: 49.5%	A combined proportional (29.3%) and additive (0.0827 mg/L) error model	GOF plots VPC bootstrap
Dostarlimab	Melhem et al. (37)	Two-compartment	Time-varying CL with a sigmoidal maximum effect (Emax) model	0.179	0.547	2.98	2.10	$\begin{array}{l} {\text{CL}}_{\text{t},\text{i}}\\ =0.179\;\times \;{\left(\text{WT}/70\right)}^{0.47}\\ \times \;{\left(\text{AGE}/64\right)}^{-0.227}\;\times \;{\left(\text{ALB}/39\right)}^{-1.01}\;\\ \times \;{\left(\text{ALT}/18\right)}^{-0.0585}\;\times \;{\left(1+0.165\right)}^{\text{ SEX}}\\ \times \text{ exp}\left(\frac{{\text{Emax}}_{\text{i}}\times T^{\text{y}}}{\text{T}50_{i}^{\text{y}}+T^{\text{y}}}\right) \end{array}$	V_C_=2.98 ×(WT/70)^0.419^× (ALB/39)^−0.153^× (1+0.162) ^SEX^	CL: 23.5% V_C_: 16.1%	A combined proportional (13.3%) and additive (2.79 mg/L) error model	GOF plots VPC

MAbs, monoclonal antibodies; PK, model pharmacokinetic model; CL, clearance; Q, inter-compartment clearance; V_C_, volume of the central compartment; V_P_, volume of distribution of the peripheral compartment; IIV, interindividual variability; PPK, population pharmacokinetics; CL_t,I_, the CL of patient i at a given time t; BBWT, baseline body weight; eGFR, estimated glomerular filtration rate; PS, performance status; RAAS, Asian race; GOF, goodness-of-fit; VPC, visual predictive check; AdjMEL, adjuvant therapy for patients with melanoma whose tumors were removed by surgical resection; RAAA, African American race; NSCLC, non-small cell lung cancer; IPI, ipililumab; CHEMO, chemotherapy coadministration; NPC, nasopharyngeal carcinoma; BALB, baseline albumin; SQ|NSQ, squamous and nonsquamous; OTH, other; GC, gastric cancer; BLDH, baseline lactate dehydrogenase; BTSIZE, baseline tumor size; CASG, with prior gastrectomy; BSA, body surface area; MIS, missing; BSLD, baseline tumor burden; ECOG, Eastern Cooperative Oncology Group; BIL, bilirubin; CANC, cancer type; MEL, melanoma; LC, lymphocyte count; TMPK, a time-dependent coefficient; TSE LCE ASE LDE, are powers adjusting the shape of the effect; CoCoν and CaCoν, baseline continuous and categorical covariates; UCC, urothelial cell carcinoma; MESO, malignant pleural mesothelioma; REF, reference; IgG, immunoglobulin G; ALT, alanine aminotransferase; BMI, body mass index; V_ss_, steady-state volume of distribution, $\text{Sigmoid}-{\text{E}}_{\text{max}}:\text{exp}\left(\frac{{\text{E}}_{\text{max}}\times {\text{T}}^{\gamma }}{{\text{T}}_{50}^{\gamma }+{\text{T}}^{\gamma }}\right)$

Among all the publications, the pharmacokinetics of anti-PD-1 mAbs were described by a two-compartment model in thirteen analyses (20–22, 28–37). The median (range) estimated CL, inter-compartmental clearance (Q), volumes of V_C_, and peripheral (V_P_) compartments were 0.249 L/day (0.179–0.290 L/day), 0.703 L/day (0.414–0.889 L/day), 3.48 L (2.98–4.46 L), and 2.78L (2.10–4.06 L), respectively. One analysis (23) developed a one-compartment model due to just trough concentrations obtained, which could not estimate the V_C_ and V_P_ (the estimated CL was 0.257 L/day, and the estimated volume of distribution was 6.8 L). Ten PPK analyses described the CL of mAbs as time-varying by a sigmoidal maximum effect model (20, 21, 28–32, 34, 35, 37), among which one analysis added time-varying covariates (20). One analysis by Wang et al. (36) used a parallel linear and nonlinear CL model to describe time-varying CL and the other three analyses (22, 23, 33) used a time-stationary CL model. The IIV of CL and V_C_ in the final model were described in fourteen and thirteen analyses, respectively. The median (range) IIV of CL reported was 30.9% (8.7%–50.8%) and V_C_ was 29.0% (4.3%–40.7%). Four analyses (28, 34, 36, 37) demonstrated that the IIV for anti-PD-1 mAbs CL and V_C_ in the final model were reduced by 10.9%–30.0% and 7.5%–21.0% compared with the base model. More details are given in **Supplementary Figure 1**. The proportional residual error model was commonly used to describe random residual variability in eight analyses (21–23, 28–32), and the median (range) proportional error across the analyses was 22.1% (2.0%–100%). The log-transformed additive error was applied in two analyses (33, 34), which were 27.2% and 25.1%. The combined proportional and additive error was applied in three analyses (35–37), among which the proportional error was 18.8%, 29.3%, and 13.3%; the additive error was 1.48, 0.0827, and 2.79 mg/L, respectively. The information about residual error was not available in one analysis (20).

The final models of the included analyses were all evaluated by internal evaluation. Several frequently used methods were: visual predictive checks (VPC) (21–23, 28–37), goodness-of-fit plots (GOF) (20–23, 28, 30, 32–37) and bootstrapping (20–23, 28, 29, 31–36). One analysis applied the posterior predictive check approach (33).

### Covariate modeling

One of the purposes of most PPK analyses was to identify potential covariates to describe the IIV of the PK of anti-PD-1 mAbs. The covariate screening process and covariates included in the final models were summarized in **Table 4**.

**Table 4:** List of tested and included covariates in the PPK models of anti-PD-1 mAbs.

MAbs	Analyses	Tested covariates	Covariate selection	Included covariates			
				CL	V_C_	Q	V_P_
Nivolumab	Bajaj et al. (28)	BW, AGE, SEX, RACE, PS, eGFR, HEPATIC, TUMOR TYPE, ADA	Backward elimination	BW, SEX, RACE, PS, eGFR	BW, SEX	–	–
Nivolumab	Hamuro et al. (21)	BW, SEX, RACE, PS, eGFR, TUMOR TYPE	Previous model (28) was used as the base model	BW, SEX, RACE, PS, eGFR, TUMOR TYPE	BW, SEX	–	–
Nivolumab	Hurkmans et al. (22)	BSA, SEX, AGE, TUMOR TYPE, PS, BW, BTSIZE, CREAT, RENAL, TP, LDH, RACE, ALB, LEUCOCYTE	Forward inclusion and backward elimination	BSA, SEX, ALB	–	–	–
Nivolumab	Osawa et al. (29)	BW, SEX, RACE, PS, eGFR, TUMOR TYPE, ALB, LDH, BTSIZE, CASG, CASG-MIS	Backward elimination	BW, SEX, RACE, PS, eGFR, TUMOR TYPE, ALB, LDH, BTSIZE, CASG, CASG-MIS	BW, SEX	–	–
Nivolumab	Wang et al. (30)	BW, PS, AGE, eGFR, ALB, TUMOR TYPE, SEX, SQ|NSQ	Retained the previous covariates (28) identified and backward elimination	BW, PS, AGE, eGFR, ALB	BW, SEX, SQ|NSQ	–	–
Nivolumab	Zhang et al. (31)	BW, SEX, RACE, PS, eGFR, TUMOR TYPE	Previous model (28) was used as the base model	BW, SEX, RACE, PS, eGFR, TUMOR TYPE	BW, SEX	–	–
Nivolumab	Zhang et al. (32)	BW, SEX, RACE, PS, eGFR, IPICO, CHEMO, TUMOR TYPE	Backward elimination	BW, SEX, RACE, PS, eGFR, IPICO, CHEMO	BW, SEX	BW	BW
Pembrolizumab	Ahamadi et al. (33)	SEX, AGE, RACE, AST, BIL, ALP, PS, eGFR, TUMOR TYPE, ALB, BTSIZE, IPI, GLU	Forward inclusion and backward elimination	SEX, PS, eGFR, TUMOR TYPE, ALB, BTSIZE, IPI	SEX, ALB, IPI	–	–
Pembrolizumab	Li et al. (34)	AGE, RACE, AST, ALT, ALP, SEX, PS, eGFR, TUMOR TYPE, ALB, BTSIZE, BIL, GLU, IgG	Forward inclusion and backward elimination	SEX, PS, eGFR, TUMOR TYPE, ALB, BTSIZE, BIL	SEX, ALB	–	–
Pembrolizumab	Li et al. (20)	BW, AGE, eGFR, ALP, AST, ALT, BIL, SEX, RACE, GLU, PS, GEOGRAPHIC LOCATION, SMOKE, ALB, BTSIZE	Retained the covariates of previous study (34)	SEX, PS, eGFR, ALB, BTSIZE, BIL	SEX, ALB	–	–
Pembrolizumab	Hurkmans et al. (23)	BSA, SEX, AGE, TUMOR TYPE, PS, BW, RENAL FUNCTION, ALB, CREAT, TP, LDH, LEUCOCYTE	Forward inclusion and backward elimination	BSA, ALB, TUMOR TYPE	LDH, MESO	–	–
Cemiplimab	Yang et al. (35)	BW, BMI, SEX, AGE, RACE, BIL, PS, ALB, LDH, TUMOR TYPE, ALT, IgG, CREATBL, CRCLBL, AST, ALP, CORTFLN, ADA	Forward inclusion and backward elimination	BW, ALB, ALT, IgG	BW, BMI	BW, ALB, ALT, IgG	BW, BMI
Camrelizumab	Wang Y et al. (36)	BW, SEX, AGE, RACE, CREAT, BIL, ALT, AST, ALB, TUMOR TYPE, ADA, PLATELETS, WBCs, APTT	Forward inclusion and backward elimination	ALB	–	BW	–
Dostarlimab	Melhem et al. (37)	BW, AGE, RACE, SEX, CRCL, RENAL, LIVER FUNCTION MARKERS, ALT, ALB, BTSIZE, GLU, RECIST, ADA	Forward inclusion and backward elimination	BW, SEX, AGE, ALB, ALT	BW, SEX, ALB	BW	–

MAbs, monoclonal antibodies; CL, clearance; V_C_, volume of the central compartment; Q, inter-compartment clearance; V_P_, volume of the peripheral compartment; BW, body weight; PS, performance status; eGFR, estimated glomerular filtration rate; ADA, anti-drug antibody; IPICO, ipililumab coadministration; CHEMO, chemotherapy coadministration; ALB, albumin; SQ|NSQ, squamous and nonsquamous; LDH, lactate dehydrogenase; BTSIZE, baseline tumor size described by the sum of long diameters of target tumor lesions; CASG, with prior gastrectomy; MIS, missing; BSA, body surface area; CREAT, Creatinine; TP, total protein; AST, aspartate aminotransferase; BIL, bilirubin; ALP, alkaline phosphatase; IPI, ipilimumab treatment status: naive or treated; GLU, glucocorticoids; ALT, alanine aminotransferase; IgG, immunoglobulin G; MESO, malignant pleural mesothelioma; BMI, body mass index; CREATBL, creatinine concentration at baseline; CRCLBL, creatinine clearance at baseline; CORTFLN, corticosteroid (yes or no); WBCs, white blood cells; APTT, activated partial thromboplastin time; RECIST, sum of diameters of measurable target lesions per immune-related (ir) Response Evaluation Criteria in Solid Tumors.

In all the publications, covariates assessed for inclusion in the model included demographic factors (body size, sex, age, and race), biological factors (renal and hepatic function, ALB, LDH, leucocyte count, total serum protein, platelets, white blood cells and activated partial thromboplastin time), antigenic mass factors (tumor type, baseline tumor size (BTSIZE) and PS), immunity factors (antidrug antibody (ADA) and IgG) and extrinsic factors (prior treatment with ipilimumab (IPI), IPICO, chemotherapy coadministration (CHEMO), with prior gastrectomy (CASG) and glucocorticoids (GLU)).

**Figure 2:**
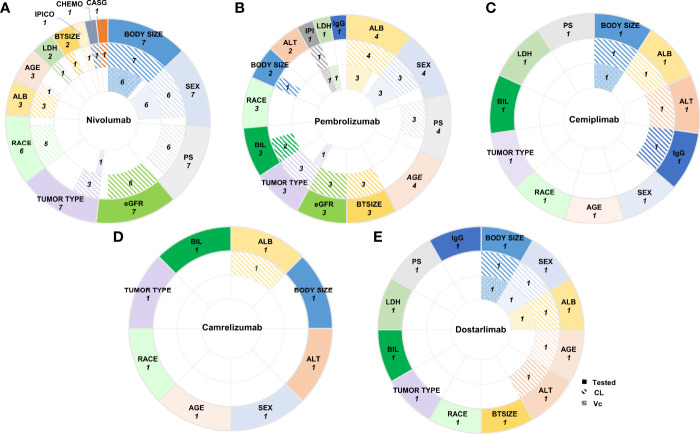
The number of PPK analyses in which covariates were tested and included in the final models. The pie charts showed the number of PPK analyses in which covariates were tested, included in the final models affecting CL or V_C_ (from outermost to innermost ring) of **(A)** Nivolumab, **(B)** Pembrolizumab, **(C)** Cemiplimab, **(D)** Camrelizumab, and **(E)** Dostarlimab. Tested Covariates for each anti-PD-1 mAbs shown here were those included in more than one final PPK models. CL, clearance; V_C_, volume of the central compartment; CASG, with prior gastrectomy; CHEMO, chemotherapy; IPICO, ipililumab coadministration; BTSIZE, baseline tumor size described by the sum of long diameters of target tumor lesions; LDH, lactate dehydrogenase; ALB, albumin; eGFR, estimated glomerular filtration rate; PS, performance status; IgG immunoglobulin G; IPI, ipililumab prior treatment status: naive or treated; ALT, alanine aminotransferase; BIL, bilirubin.

Among the above covariates, of which included in the final model were usually identified using the stepwise covariate method involving forward addition and backward elimination. The frequently reported as significant covariates on CL were the effects of body size (nivolumab 7/7, pembrolizumab 1/4, cemiplimab 1/1, dostarlimab 1/1) (21–23, 28–32, 35, 37), ALB (nivolumab 3/7, pembrolizumab 4/4, cemiplimab 1/1, camrelizumab 1/1, dostarlimab 1/1) (20, 22, 23, 29, 30, 33–37), sex (nivolumab 6/7, pembrolizumab 3/4, dostarlimab 1/1) (20–22, 28, 29, 31–34, 37), eGFR (nivolumab 6/7, pembrolizumab 3/4) (20, 21, 28–34), PS (nivolumab 6/7, pembrolizumab 3/4) (20, 21, 28–34), tumor type (nivolumab 3/7, pembrolizumab 3/4) (21, 23, 29, 31, 33, 34), race (nivolumab 5/7) (21, 28, 29, 31, 32), and BTSIZE (nivolumab 1/7, pembrolizumab 3/4) (20, 29, 33, 34). In addition, a small number of covariates related to CL have been reported, such as bilirubin (BIL) (20, 34), IPICO (32), IPI (33), CHEMO (32), CASG (29), age (30, 37), LDH (29), alanine aminotransferase (ALT) (35, 37), and IgG (35). The effect on V_C_ were commonly described by sex (nivolumab 6/7, pembrolizumab 3/4, dostarlimab 1/1) (20, 21, 28–34, 37) and BW (nivolumab 6/7, cemiplimab 1/1, dostarlimab 1/1) (21, 28–32, 35, 37) as the covariates, and a few analyses also added ALB (20, 33, 34, 37), tumor type (23, 30), IPI (33) and LDH (23). Covariates affecting Q and V_P_ have rarely been investigated (**Figure 2**).

### Covariate effects

**Figure 3:**
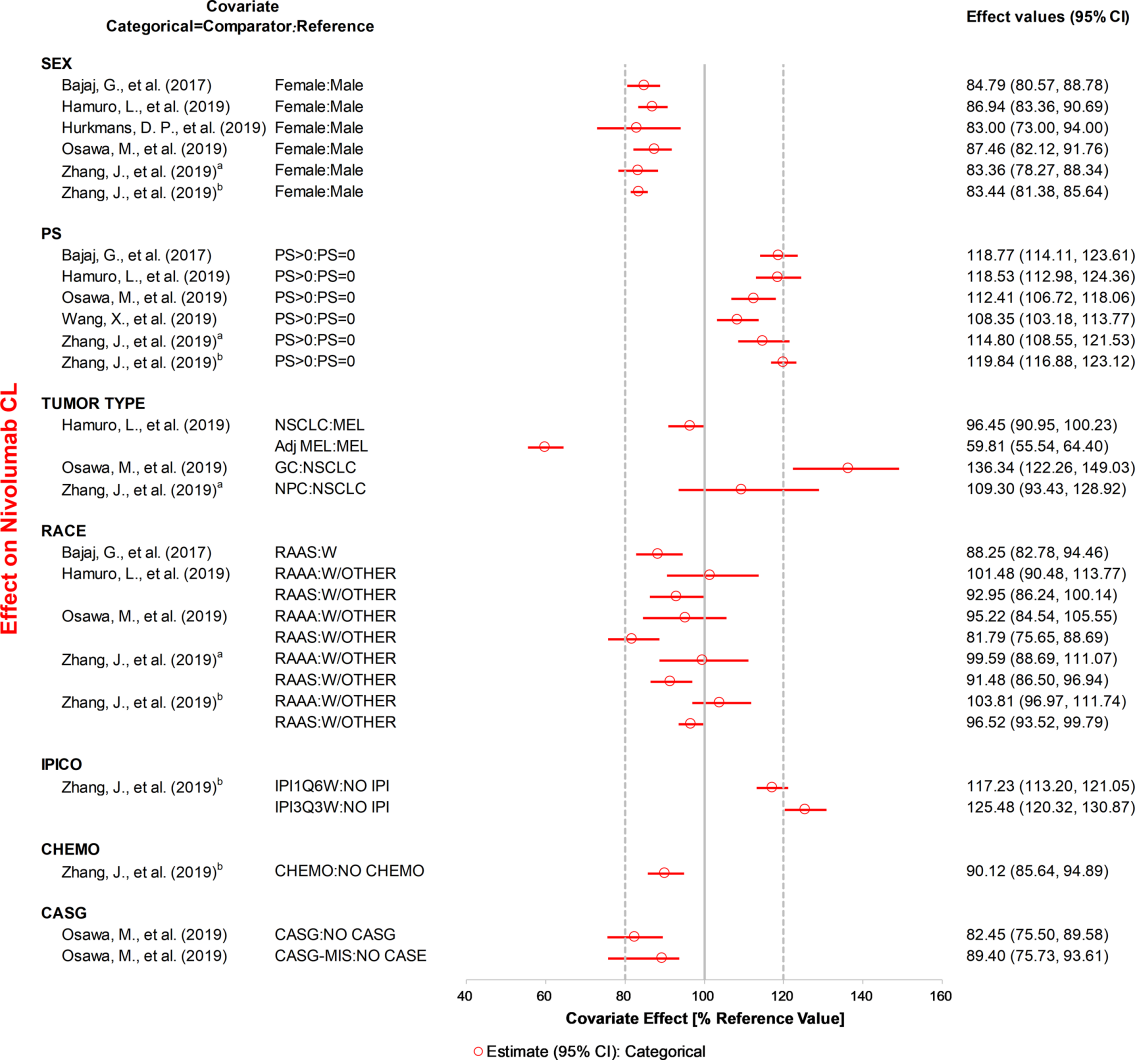
The effects of included categorical covariates on CL of nivolumab. Categorical covariate effects (95% confidence interval [CI]) are represented by open symbols (horizontal lines). The typical value of clearance in each study was considered to be 1. The effect of each covariate for clearance is displayed by the ratio of clearance in the range of each covariate to the typical clearance value. PS, performance status; IPICO, ipilimumab coadministration; CHEMO, chemotherapy; CASG, with prior gastrectomy. ^a^Reference (31), ^b^Reference (32).

**Figure 4:**
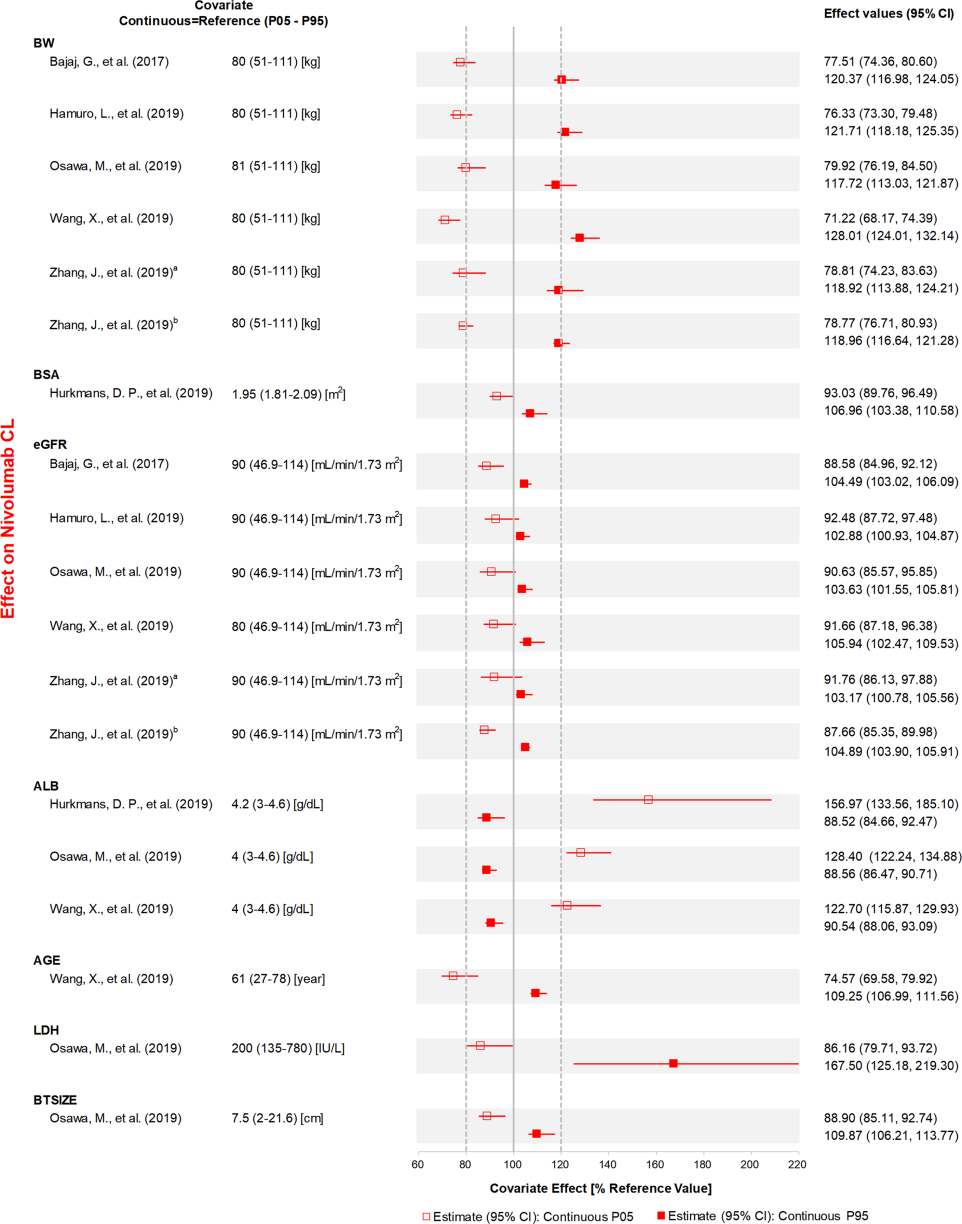
The effects of included continuous covariates on CL of nivolumab. Continuous covariate effects (95% CI) at the 5th/95th percentiles of the covariate are represented by the end of horizontal boxes (horizontal lines). The effect of each covariate for clearance is displayed by the ratio of clearance in the range of each covariate to the typical clearance value. BW, body weight; BSA, body surface area; eGFR, estimated glomerular filtration rate; ALB, albumin; LDH, lactate dehydrogenase; BTSIZE, baseline tumor size described by the sum of long diameters of target tumor lesions; P05, 5th percentile; P95, 95th percentile. ^a^Reference (31), ^b^Reference (32).

The effects of all covariates on CL and V_C_ included in the final model were assessed quantitatively (**Figures 3**, **4**, **Supplementary Figure 2** and **Supplementary Table 1**).

ALB, BW, specific tumor type, sex, and PS were the most studied covariates that had a significant influence on CL. The CL of anti-PD-1 mAbs increased with a decrease in ALB, and the covariate effect was significant in ten analyses (20, 22, 23, 29, 30, 33–37), with a median effect ranging from 22.7 to 67.3%. Eight analyses (21, 28–32, 35, 37) showed that the CL of anti-PD-1 mAbs increased with BW, with the median effect ranging from 21.1 to 29.8%. Three analyses (21, 23, 29) indicated that tumor type had a greater than 20% effect on the CL of nivolumab (median effect 36.3–40.7%) and pembrolizumab (median effect 29.0%). The CL of mAbs was lower in females compared with males (used as the reference), and the change in CL was statistically significant in five analyses (20, 22, 31, 34, 37), with the 95% CI of the median effect ranging from 22.7 to 27.0%. Four analyses (21, 28, 31, 32) found that the CL was higher in patients with PS >0 than in patients with PS of 0, with the 95% CI of median effect ranging from 21.5 to 24.3%. Two analyses (30, 37) reported that age had a significant influence on CL. However, one analysis (30) indicated that the CL of nivolumab decreased 26.5% at the low extreme of age. In contrast, another analysis (37) found that the lower age group had a higher CL of dostarlimab (21.6%). Other covariates investigated only in one study, such as LDH, IgG, and IPICO, were also considered to have significant effects on CL. LDH (29) and IgG (35) had the most positive effects on CL of nivolumab (median effect of 67.5%) and cemiplimab (median effect of 31.1%), respectively. The CL of nivolumab was greater than 25.4% for ipilimumab 3 mg/kg every 3 weeks compared with monotherapy (32). The effects of other covariates that were within the 20% boundaries were race, CHEMO, body surface area (BSA), eGFR, BTSIZE, IPI, BIL, and ALT, suggesting these covariates may not have any clinical significance.

Eight analyses (21, 28–32, 35, 37) found that the V_C_ of anti-PD-1 mAbs increased with an increase in BW, and the median magnitude of this effect was 21.5–46.2%. BMI was also an influential covariate with a decrease of 35.5% and an increase of 38.5% in V_C_, at high and low extremes of BMI, respectively (35). Hurkmans et al. (23) reported that the patients with MESO had lower V_C_ of pembrolizumab compared with other tumor types (median effect of 42.0%). The effects of other covariates on V_C_ within the 20% boundaries were sex, ALB, LDH, and IPI.

## Discussion

There has been continuous interest in studying the PK of anti-PD-1 mAbs during the past few years, and several studies have attempted to identify the sources of variability of anti-PD-1 mAbs through PPK and apply the prediction ability of the model to the subsequent studies. This review summarizes the knowledge regarding the PPK modeling of anti-PD-1 mAbs. To date, fourteen analyses have been published on the PPK model of anti-PD-1 mAbs in patients with multiple tumor types and races. Most analyses described the PK of anti-PD-1 mAbs using the two-compartment model with time-varying CL. There was higher IIV on the PK parameter of anti-PD-1 mAbs, so the relevant PPK analyses explored the possible influencing factors of pharmacokinetic variation among patients. Currently, the covariates that were included in the final model most frequently were ALB, BW, sex, eGFR, PS, tumor type, and race. The covariates that had significant effects on CL larger than 20% were ALB, BW, specific tumor type (GC, urothelial cell cancer (UCC)), sex, PS, LDH, IgG, and IPICO. The variability in V_C_ was mostly explained by BW and sex, and BW had a significant effect greater than 20%. Most models have applied 2–3 internal evaluation methods for evaluating the performance of models, such as GOF plots, VPC, and bootstrap analysis.

In all these publications, patients were administered by intravenous infusion, which was the most common route of mAb administration (38). Following intravenous administration, the concentration-time profile of mAbs often follows a bi-exponential decline, which can be best described using a two-compartment pharmacokinetic model with a zero-order infusion (39). The analysis employing real-world data by Hurkmans et al. (23) used a one-compartment model, as the serum sampling of patients obtained was the trough levels of pembrolizumab that could not estimate Q and V_P_ adequately. For the real-world analyses, the difficulty of obtaining dense blood samples is universal. The PPK analysis of nivolumab proposed that to build the two-compartment model, V_P_ can be assumed to be equal to V_C_ (22). Time-varying CL has been recently recognized in drug labels for anti-PD-1 mAbs (20), and eleven PPK analyses (20, 21, 28–32, 34–37) in our review confirmed that the incorporation of time-varying CL resulted in a statistically significant improvement in the GOF (28). However, there is no clear mechanistic understanding of the time-varying CL of mAbs (28).

Several analyses (19, 28) proposed a hypothesis that the decrease in CL during treatment may be associated with the corresponding decrease in the rate of cachexia (40). Cancer patients with cachexia consume mAbs as a source of protein in the case of metabolic imbalance. Therefore, cachexia syndrome can be reversed with effective treatment during the improvement of disease status, which results in a CL reduction of mAbs. However, whether CL is related to efficacy still needs specific research. Another potential mechanism proposed by Liu et al. shows that some tumor cells can produce protease to cleave antibodies (19), which serves as a pathway to avoid host immune surveillance. Further research is still needed to prove the mechanism.

It was reported that the higher IIV on the PK parameter of anti-PD-1 mAbs was observed in the base model without covariates. Therefore, the relevant PPK analyses explored the individual factors of PK variability to reduce IIV to improve the estimation accuracy. As shown in **Supplementary Figure 1**, four analyses included in our review provided IIV on CL and V_C_ in the base models, which were 46.0% (29.2%–57.0%) and 30.8% (17.4–44.4%), respectively. Further, the IIV of CL and V_C_ in the final model of all the PPK analyses were 30.9% (8.7%–50.8%) and 29.0% (4.32%–40.7%), respectively. The common sources of PK variability were the following possible factors: demographic factors (BW, sex, age, and race), biological factors (ALB, LDH, eGFR, BIL, and ALT), antigenic mass factors (tumor type, PS, and BTSIZE), immunity factors (IgG and ADA), and extrinsic factors (IPICO, CHEMO, IPI, and CASG).

In all PPK studies of anti-PD-1 mAbs, twelve analyses tested covariates of body size, of which ten analyses included it in the final model. Body size was usually investigated as the continuous covariate in the final model, except in two studies of pembrolizumab (33, 34), which used PK parameters to be allometrically scaled based on BW. Eight studies demonstrated that BW had a significant effect on CL (median effect 21.1%–29.8%) and V_C_ (median effect 21.5%–46.2%). Two analyses reported that BSA may have no clinical significance on CL due to covariate effect <20%. Larger individuals had higher CL and V_C_ due to a greater volume of plasma and interstitial fluid when compared with smaller individuals (23, 27, 28, 35–37). This was supported by the argument that PK parameters such as CL and V_C_ are usually functions of body size, which correlates to physical volume (27, 41). One study found that obesity has significant negative effects on lymphatic transport (42), which also may influence the rate and extent of mAb distribution in tissues.

Fourteen analyses tested the effect of sex on PK, ten of which included it as a covariate in the final model. They found that sex had a significant effect on the CL of anti-PD-1 mAbs with the 95% CI of covariate effect ranging from 22.7% to 27.0%, and CL and V_C_ were lower in females than in males (21, 22, 28, 29, 31–34). The differences may stem from multiple potential reasons, including different lymph flow rates, which may affect distribution; different target levels, which may affect target mediated drug disposition; different endocytosis, which may influence distribution; and different FcγR expression levels and immunogenicity, which may be related to the CL of mAbs (27, 43). However, to our knowledge, there was no consistent physiological explanation to clarify this confounding factor. So far, sex has never led to the dose adjustment of anti-PD-1 mAbs.

Age was also the factor frequently tested in the ten anti-PD-1 mAb PPK publications. However, only two analyses of nivolumab and dostarlimab reported that age had a significant influence on CL. One analysis (30) indicated that the CL of nivolumab was decreased by 26.5% at the low extreme of age, whereas another analysis (37) found that the lower age had a higher CL of dostarlimab (21.6%). The effect of age remains questionable in adults with cancer, which may explain why age is considered to have no clinical relevance, despite statistical significance. To our knowledge, no convincing explanation has been reported for this effect, and no specific dosing adjustment at age with mAbs has been proposed so far (44).

The influence of race was investigated in eleven publications, five of which included it as a categorical covariate in the final model. However, race had no clinically meaningful effect on the PK of anti-PD-1 mAbs. The impact mechanism of race on the PK of mAbs remains unclear, even if gene/target protein expression, tumor burden, disease progression, FcγR polymorphisms, and body size of different races were different (45).

Ten analyses that included ALB as a test covariable identified that it was an important covariate, and increased ALB levels were indicative of decreased CL of anti-PD-1 mAbs (median effect of 22.7%–67.3%). On the one hand, several analyses have considered that ALB and mAbs are recycled by FcRn in a non-competitive manner. So, high ALB levels reflect high FcRn activity, and the CL of mAbs is low by recycling. On the other hand, in most publications, researchers propose that hypoalbuminemia is linked to cachexia or might be caused by inflammation, so ALB can be accurately used as an indicator of the metabolic state and the high tumor burden in tumor patients (38). In these cases, the CL of mAbs may increase with increased protein catabolism or targeted-mediated elimination (44). However, its specific impact mechanism remains to be studied.

Five analyses investigated the effect of LDH on the PK of anti-PD-1 mAbs, two of which included it in the final model. One analysis reported that LDH had a positive effect on the CL of nivolumab (median effect of 67.5%). Other research included LDH as the covariate on V_C_. However, LDH may not have any clinical significance on V_C_, as the effect in V_C_ was found to be <20%. LDH level was found to be a key metabolic hallmark of cancer cells (20). Higher LDH levels may also demonstrate more advanced disease, with a higher likelihood of cancer cachexia (23). However, the effect of serum LDH levels has rarely been included in other PPK models. Further studies are needed to confirm the covariate effect and the influential mechanism of LDH.

eGFR was tested in ten analyses, nine of which included the covariate in the final model. However, eGFR was unlikely to be a clinically relevant covariate, as the estimated change in CL in patients with renal impairment was within 20%. The hepatic functions (BIL and ALT) affecting the PK of anti-PD-1 mAbs were included in three analyses. However, renal or hepatic impairment had no clinically relevant effect on mAbs with covariate effects of <20% (46). Theoretically, the large size of anti-PD-1 mAbs is expected to prevent them from being filtered through the glomeruli of the kidney and hepatic elimination (28).

Thirteen analyses tested the effect of different tumor types on PK, seven of which included it as a categorical covariate in the final model. Osawa et al. (29) found that the baseline CL of nivolumab in patients with GC was 36.3% greater than in patients with NSCLC in the second line or subsequent lines of treatment. Hurkmans et al. (23) found that the CL of pembrolizumab in UCC patients was 29.0% higher than in patients with other cancer types. Hamuro et al. (21) reported that for patients with MEL whose tumors were removed by surgical resection with adjuvant therapy (AdjMEL), the geometric mean nivolumab CL was 40.2% lower at baseline and did not vary with time and 20% lower at steady state compared with patients with MEL (21). Four other analyses reported similar CL estimates in patients with NPC, NSCLC, and MEL.

Other covariates related to antigen mass, such as PS and BTSIZE, may be considered factors affecting the PK of mAbs (44). Twelve analyses tested the effect of PS on CL, nine of which included it as a covariate in the final model. CL in patients with PS >0 appeared higher compared to patients with a PS of 0 (28). Four analyses showed that PS had a significant effect on CL, with the 95% CI of the median effect ranging from 21.5% to 24.3%. Indeed, patients with high tumor burden or PS >0 may have cachexia, which leads to increased protein catabolism (44). Four analyses included BTSIZE and reported that it had no clinical significance (covariate effect <20%).

There were some covariates related to immunity, such as IgG and ADA, mentioned in a few publications. Three analyses tested the effect of IgG on PK, one of which (35) included it in the final model. It was found that high baseline IgG may be related to increased CL of cemiplimab with a covariate effect larger than 20%. Four analyses assessed the effect of ADA on the PK of anti-PD-1 mAbs, none of which included it as the final model without a specific explanation. Although few reports have reported that ADA has a clinical impact on anti-PD-1 mAbs in oncology, it may lead to secondary treatment failure and an increased incidence of adverse drug reactions (44). Therefore, more systematic and extensive research on its impact is needed.

In addition to the above intrinsic factors, three analyses analyzed the effects of extrinsic factors, such as concomitant medications (IPICO, CHEMO), and prior treatment status (IPI, CASG). However, the covariates may not be clinically relevant except for IPICO. Zhang et al. (32) reported that IPICO 3 mg/kg every 3 weeks resulted in a 25.5% increase in nivolumab CL, and the decrease of nivolumab CL over time was greater. The underlying mechanism of the interactions between mAbs in combination is unknown, with few published examples of interactions between combined mAbs and PK.

Overall, ALB, BW, specific tumor type, sex, and PS had a significant influence on CL that were the most studied covariates, with the covariate effect (95% CI) larger than 20%. The effects of LDH, IPICO, and IgG in CL were also more than 20%, but there were few studies. Therefore, more studies are needed to verify these covariates. BW was the primary covariate on V_C_, with a covariate effect greater than 20%.

Currently, PPK modeling of anti-PD-1 mAbs faces several challenges. As can be seen from **Supplementary Figure 1**, there were still 70.0%–81.1% and 79.0%–92.5% unexplained IIV on the CL and V_C_ of anti-PD-1 mAbs from the base model in four analyses. This meant that the identified covariates affecting the PK of anti-PD-1 mAbs were insufficient to explain most of the IIV. Unknown or new covariates related to the physiology of anti-PD-1 mAbs deserve to be explored in the future to decrease non-negligible IIV. Additionally, the confounding interplay between covariates hampers the understanding of the mechanisms of the influence of covariates. There is an urgent need to investigate and explore the physiologic and pathophysiologic mechanisms of anti-PD-1 mAbs.

As for the application of PPK models, some analyses used relevant PPK models to improve the ease of administration by predicting and comparing anti-PD-1 exposure of several administration regimens, including the flat dose instead of dose based on BW (47–50), less frequent treatment (51–54), and infusion times (55). It has been shown that the improvement of dosing regimens offers the advantages of increasing convenience, easy preparation, reducing the risk of administration error, minimizing waste, and improving compliance and patient adherence (50). Other studies predicted the anti-PD-1 exposure of each patient for the analyses of exposure–response (E–R) relationships across different population types by PPK models (28, 56). Moreover, some researchers (19, 21, 57) have also used the PPK models to predict the CL of anti-PD-1 mAbs in patients for individualization and found that time-varying CL might be useful as a biomarker for therapy success (58). Although the PPK models of anti-PD-1 mAbs were validated internally using the same dataset for constructing the model, no external validations have been conducted using an independent dataset. External validation is the most stringent approach for model evaluation. Therefore, to allow the PPK models to be successfully applied to clinical practice to obtain individualized doses, further studies must performed to validate these models.

However, there are some limitations to be mentioned. Firstly, due to the lack of details in some publications, a small amount of model information was missing in this review. Secondly, most of the PPK analyses of anti-PD-1 mAbs were based on the models established by clinical trial data, and there were only two analyses that were based on real-world data. Hence, most of the study population was a representative sample of the target population. Thirdly, even if covariates were investigated in PPK analyses, there is a large heterogeneity of covariate availability or investigation among publications. In the end, most analyses were for nivolumab and pembrolizumab due to their earlier marketing. There is currently no PPK analysis on tislelizumab, toripalimab, sintilimab, prolgolimab, penpulimab, and zimberelimab. Therefore, the models of these mAbs are not provided in this review. Moreover, since the publications included were all in English, it may have omitted the research published in other languages.

## Conclusion

Anti-PD-1 mAbs have become a considerable component of cancer immunotherapy for a growing number of tumor patients. This review provides the parameters used for constructing anti-PD-1 mAb models, key features of these models, and established covariate relationships in detail. This can allow for a deeper knowledge of anti-PD-1 mAb pharmacokinetics, provide a reference for building PPK models of other anti-PD-1 mAbs, and identify areas requiring additional research to facilitate the application of PPK models. Because there is still variability that cannot be ignored, further research in actual clinical practice for personalized PD-1 therapy should be conducted and new or controversial potential covariates should be tested in the future. Before applying the model to move on to the next step of the research, the previously released model should be externally evaluated, and the prediction performance of various models should be compared to determine the applicability of using relevant models.

## Data availability statement

The original contributions presented in the study are included in the article/**Supplementary material**. Further inquiries can be directed to the corresponding authors.

## Author contributions

The contributions were made by all authors. JS and YL contributed to the first draft of the manuscript. LH and JH revised the initial manuscript. YF and XR edited the manuscript. All authors read the article and approved the final version.

## Funding

The study was supported by the Bethune Charitable Foundation of Pharmaceutical Research Capacity Building Project (B-19-H-20200622).

## Conflict of interest

The authors declare that the research was conducted in the absence of any commercial or financial relationships that could be construed as a potential conflict of interest.

## Publisher’s note

All claims expressed in this article are solely those of the authors and do not necessarily represent those of their affiliated organizations, or those of the publisher, the editors and the reviewers. Any product that may be evaluated in this article, or claim that may be made by its manufacturer, is not guaranteed or endorsed by the publisher.

## Supplementary material

Supplementary Figure 1 The percentage of explained and unexplained CL **(A)** and V_C_ **(B)** variability in the base and final models for nivolumab, pembrolizumab, dostarlimab and camrelizumab.

Supplementary Figure 2 The effects of included covariates on CL of **(A)** pembrolizumab, **(B)** dostarlimab, **(C)** cemiplimab and **(D)** camrelizumab.

Supplementary Table 1 The effects of included covariates on V_C_ of nivolumab, pembrolizumab, dostarlimab and cemiplimab.
