# Supplementary material for: Population pharmacokinetic models of anti-PD-1 mAbs in patients with multiple tumor types: A systematic review
Source: Front Immunol. 2022 Aug 2;13:871372. doi: 10.3389/fimmu.2022.871372 (PMC9379304; doi:10.3389/fimmu.2022.871372)
Supplement: Supplementary Figure 1 — The percentage of explained and unexplained CL (A) and VC (B) variability in the base and final models for nivolumab, pembrolizumab, dostarlimab and camrelizumab. [file DataSheet_1.pdf]

Supplementary materials

Supplementary Figure 1. The percentage of explained and unexplained CL(A) and V_C(B) variability in the base and final models for nivolumab, pembrolizumab, dostarlimab and camrelizumab.

Supplementary Figure 2. The effects of included covariates on CL of (A) pembrolizumab, (B) dostarlimab, (C) cemiplimab and (D) camrelizumab.

Supplementary Table 1. The effects of included covariates on V_C of nivolumab, pembrolizumab, dostarlimab and cemiplimab.

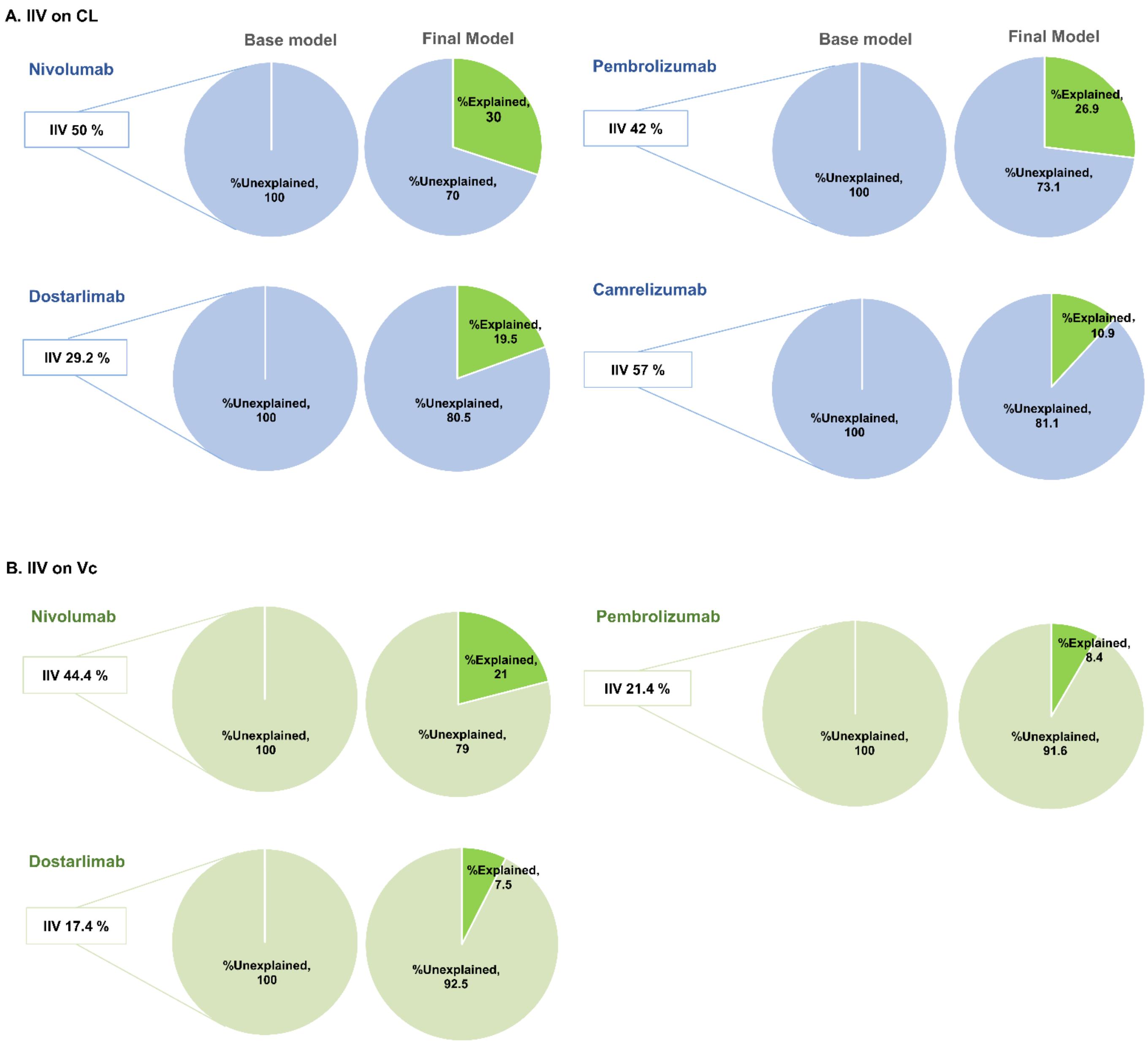

Supplementary Figure 1. The percentage of explained and unexplained CL(A) and V_C(B) variability in the base and final models for nivolumab, pembrolizumab, dostarlimab and camrelizumab. *The covariates included in the final model can minimize the interindividual variability in the base model. The figure shows that four anti-PD-1 mAbs' final model reduced the percentage of unexplainable variation by including CL and* V_C *covariates. As the covariates affecting cemiplimab* V_C *were not included in the final model, the figure of interindividual variability of cemiplimab* V_C *was not presented. IIV interindividual variability, CL clearance,* V_C *central volume of distribution*

A

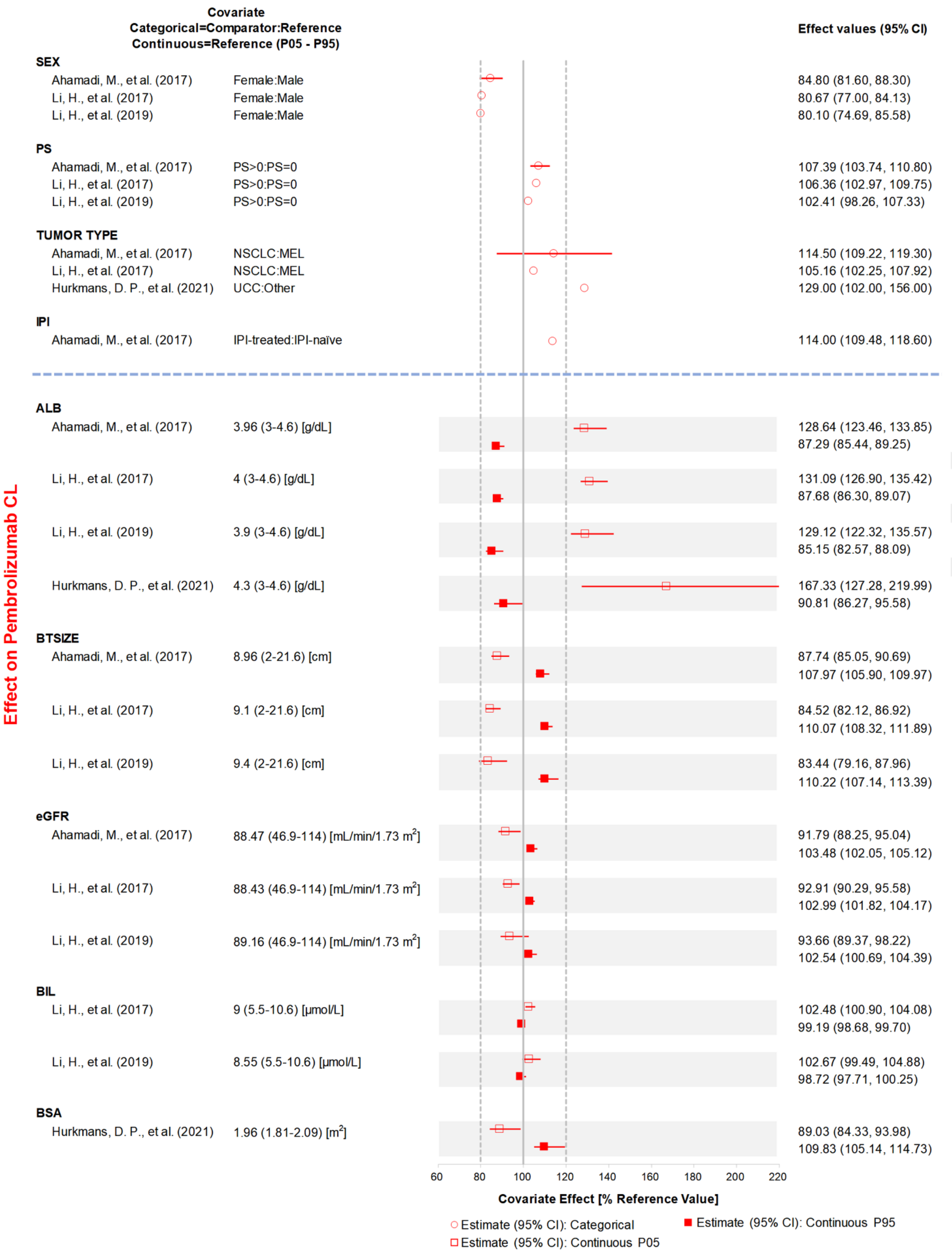
Covariate
Categorical=Comparator:Reference
Continuous=Reference (P05 - P95)
Effect values (95% CI)
SEX
Ahamadi, M., et al. (2017) Female:Male 84.80 (81.60, 88.30)
Li, H., et al. (2017) Female:Male 80.67 (77.00, 84.13)
Li, H., et al. (2019) Female:Male 80.10 (74.69, 85.58)
PS
Ahamadi, M., et al. (2017) PS>0:PS=0 107.39 (103.74, 110.80)
Li, H., et al. (2017) PS>0:PS=0 106.36 (102.97, 109.75)
Li, H., et al. (2019) PS>0:PS=0 102.41 (98.26, 107.33)
TUMOR TYPE
Ahamadi, M., et al. (2017) NSCLC:MEL 114.50 (109.22, 119.30)
Li, H., et al. (2017) NSCLC:MEL 105.16 (102.25, 107.92)
Hurkmans, D. P., et al. (2021) UCC:Other 129.00 (102.00, 156.00)
IPI
Ahamadi, M., et al. (2017) IPI-treated:IPI-naïve 114.00 (109.48, 118.60)
ALB
Ahamadi, M., et al. (2017) 3.96 (3-4.6) [g/dL] 128.64 (123.46, 133.85) 87.29 (85.44, 89.25)
Li, H., et al. (2017) 4 (3-4.6) [g/dL] 131.09 (126.90, 135.42) 87.68 (86.30, 89.07)
Li, H., et al. (2019) 3.9 (3-4.6) [g/dL] 129.12 (122.32, 135.57) 85.15 (82.57, 88.09)
Hurkmans, D. P., et al. (2021) 4.3 (3-4.6) [g/dL] 167.33 (127.28, 219.99) 90.81 (86.27, 95.58)
BTSIZE
Ahamadi, M., et al. (2017) 8.96 (2-21.6) [cm] 87.74 (85.05, 90.69) 107.97 (105.90, 109.97)
Li, H., et al. (2017) 9.1 (2-21.6) [cm] 84.52 (82.12, 86.92) 110.07 (108.32, 111.89)
Li, H., et al. (2019) 9.4 (2-21.6) [cm] 83.44 (79.16, 87.96) 110.22 (107.14, 113.39)
eGFR
Ahamadi, M., et al. (2017) 88.47 (46.9-114) [mL/min/1.73 m2] 91.79 (88.25, 95.04) 103.48 (102.05, 105.12)
Li, H., et al. (2017) 88.43 (46.9-114) [mL/min/1.73 m2] 92.91 (90.29, 95.58) 102.99 (101.82, 104.17)
Li, H., et al. (2019) 89.16 (46.9-114) [mL/min/1.73 m2] 93.66 (89.37, 98.22) 102.54 (100.69, 104.39)
BIL
Li, H., et al. (2017) 9 (5.5-10.6) [μmol/L] 102.48 (100.90, 104.08) 99.19 (98.68, 99.70)
Li, H., et al. (2019) 8.55 (5.5-10.6) [μmol/L] 102.67 (99.49, 104.88) 98.72 (97.71, 100.25)
BSA
Hurkmans, D. P., et al. (2021) 1.96 (1.81-2.09) [m2] 89.03 (84.33, 93.98) 109.83 (105.14, 114.73)
Effect on Pembrolizumab CL
60 80 100 120 140 160 180 200 220
Covariate Effect [% Reference Value]
Estimate (95% CI): Categorical
Estimate (95% CI): Continuous P05
Estimate (95% CI): Continuous P95

B

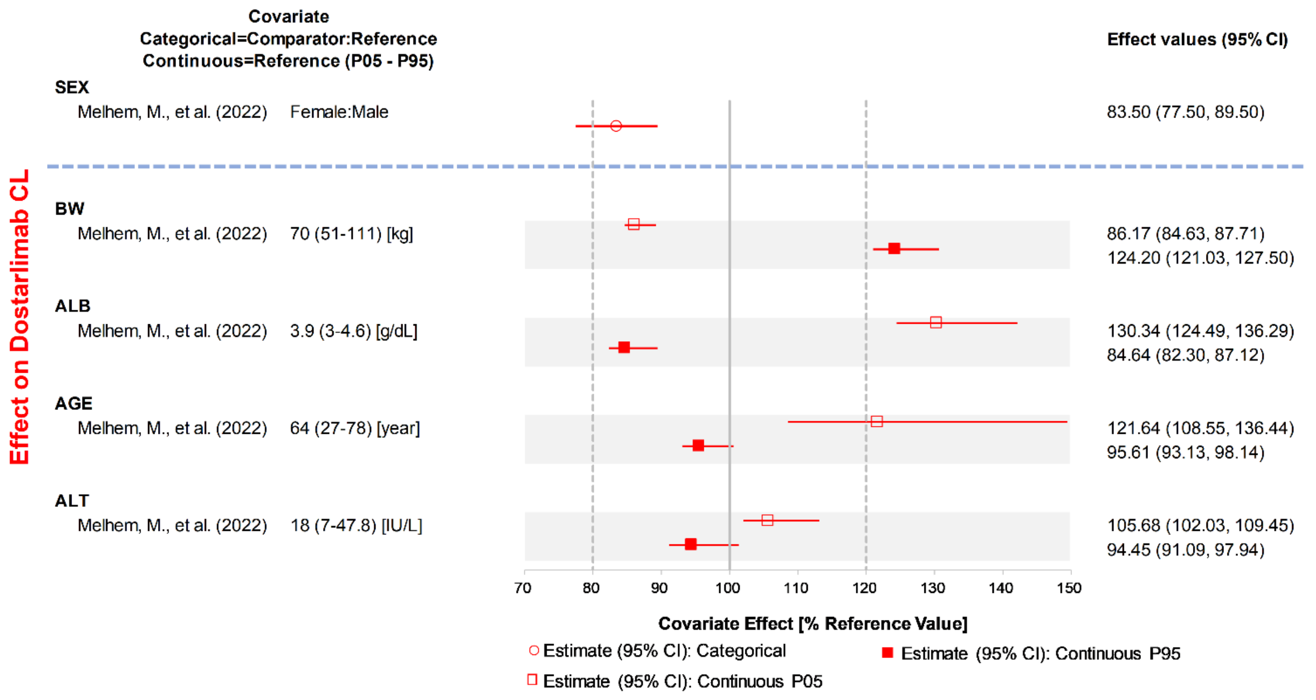
Covariate
Categorical=Comparator:Reference
Continuous=Reference (P05 - P95)
Effect values (95% CI)
Effect on Dostarlimab CL
SEX
Melhem, M., et al. (2022) Female:Male
83.50 (77.50, 89.50)
BW
Melhem, M., et al. (2022) 70 (51-111) [kg]
86.17 (84.63, 87.71)
124.20 (121.03, 127.50)
ALB
Melhem, M., et al. (2022) 3.9 (3-4.6) [g/dL]
130.34 (124.49, 136.29)
84.64 (82.30, 87.12)
AGE
Melhem, M., et al. (2022) 64 (27-78) [year]
121.64 (108.55, 136.44)
95.61 (93.13, 98.14)
ALT
Melhem, M., et al. (2022) 18 (7-47.8) [IU/L]
105.68 (102.03, 109.45)
94.45 (91.09, 97.94)
70
80
90
100
110
120
130
140
150
Covariate Effect [% Reference Value]
Estimate (95% CI): Categorical
Estimate (95% CI): Continuous P95
Estimate (95% CI): Continuous P05

C

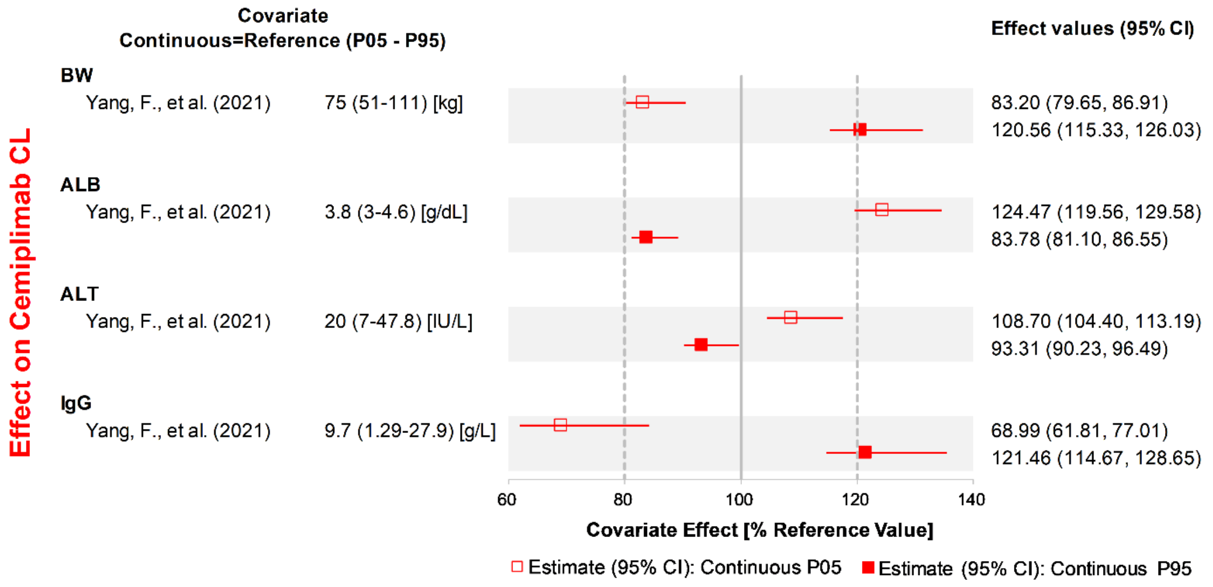
Covariate
Continuous=Reference (P05 - P95)
Effect values (95% CI)
Effect on Cemiplimab CL
BW
Yang, F., et al. (2021) 75 (51-111) [kg]
83.20 (79.65, 86.91)
120.56 (115.33, 126.03)
ALB
Yang, F., et al. (2021) 3.8 (3-4.6) [g/dL]
124.47 (119.56, 129.58)
83.78 (81.10, 86.55)
ALT
Yang, F., et al. (2021) 20 (7-47.8) [IU/L]
108.70 (104.40, 113.19)
93.31 (90.23, 96.49)
IgG
Yang, F., et al. (2021) 9.7 (1.29-27.9) [g/L]
68.99 (61.81, 77.01)
121.46 (114.67, 128.65)
60
80
100
120
140
Covariate Effect [% Reference Value]
Estimate (95% CI): Continuous P05
Estimate (95% CI): Continuous P95

D

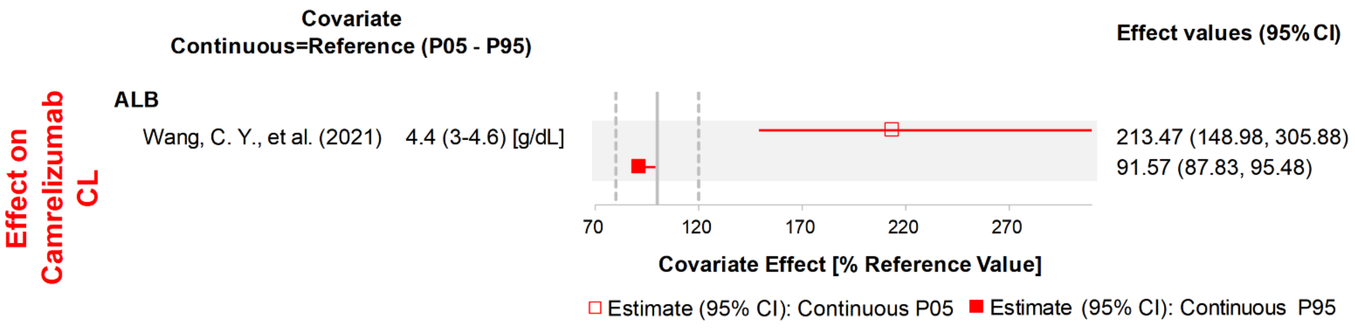
Covariate
Continuous=Reference (P05 - P95)
Effect values (95% CI)
Effect on Camrelizumab CL
ALB
Wang, C. Y., et al. (2021) 4.4 (3-4.6) [g/dL]
213.47 (148.98, 305.88)
91.57 (87.83, 95.48)
70
120
170
220
270
Covariate Effect [% Reference Value]
Estimate (95% CI): Continuous P05
Estimate (95% CI): Continuous P95

Supplementary Figure 2. The effects of included covariates on CL of (A) pembrolizumab, (B) dostarlimab, (C) cemiplimab and (D) camrelizumab. *Categorical covariate effects (95% confidence interval [CI]) are represented by open symbols (horizontal lines). Continuous covariate effects (95% CI) at the 5th/95th percentiles of the covariate are represented by the end of horizontal boxes (horizontal lines). The typical value of clearance in each study was considered to be 1. The effect of each covariate for clearance is displayed by the ratio of clearance in the range of each covariate to the typical clearance value. PS performance status, IPI ipililumab prior treatment status: naive or treated, BTSIZE baseline tumor size described by the sum of long diameters of target tumor lesions, eGFR estimated glomerular filtration rate, BIL bilirubin, BSA body surface area, ALB albumin, ALT alanine aminotransferase, BW body weight, IgG immunoglobulin G, P05 5th percentile, P95 95th percentile*

Supplementary Table 1. The effects of included covariates on V_C of nivolumab, pembrolizumab, dostarlimab and cemiplimab.

anti-PD-1 mAbs	Continuous covariate	Reference	Reference value (P05-P95)	Estimate (95%CI): P05	Estimate (95%CI): P95	Categorical covariate	Reference	Comparator: Reference	Estimate (95%CI)
Nivolumab	BW	Bajaj, G., et al. (2017)	80 (51-111) [kg]	76.43 (73.6, 79.34)	121.59 (118.33, 124.99)	SEX	Bajaj, G., et al. (2017)	Female: Male	85.9 (81.87, 89.58)
		Hamuro, L., et al. (2019)	80 (51-111) [kg]	78.07 (74.66, 81.62)	119.74 (115.92, 123.68)		Hamuro, L., et al. (2019)	Female: Male	85.81 (81.79, 90.03)
		Osawa, M., et al. (2019)	80 (51-111) [kg]	82.47 (79.59, 85.38)	115.05 (112.18, 118.06)		Osawa, M., et al. (2019)	Female: Male	82.78 (78.66, 86.85)
		Wang, X., et al. (2019)	80 (51-111) [kg]	75.82 (72.9, 78.85)	122.31 (118.87, 125.85)		Wang, X., et al. (2019)	Female: Male	90.3 (86.18, 94.63)
		Zhang, J., et al. (2019)[a]	80 (51-111) [kg]	71.67 (68.54, 74.9)	127.42 (123.4, 131.62)		Zhang, J., et al. (2019)[a]	Female: Male	87.63 (82.94, 92.24)
		Zhang, J., et al. (2019)[b]	80 (51-111) [kg]	78.63 (77.05, 80.24)	119.11 (117.37, 120.88)		Zhang, J., et al. (2019)[b]	Female: Male	85.13 (82.78, 87.63)
						TUMOR TYPE	Wang, X., et al. (2019)	SQ+NSQ: Other	85.21 (82.07, 88.48)
Pembrolizumab	ALB	Ahamadi, M., et al. (2017)	3.96 (3-4.6) [(g/dL)]	105.94 (103.16, 108.81)	96.93 (95.55, 98.34)	SEX	Ahamadi, M., et al. (2017)	Female: Male	86.6 (84.2, 89.1)
		Li, H., et al. (2017)	4 (3-4.6) [(g/dL)]	106.72 (104.26, 109.24)	96.89 (95.8, 97.99)		Li, H., et al. (2017)	Female: Male	85.32 (82.54, 87.98)
		Li, H., et al. (2019)	3.9 (3-4.6) [(g/dL)]	107.28 (104.23, 110.48)	95.67 (93.92, 97.43)		Li, H., et al. (2019)	Female: Male	84.26 (80.24, 88.64)
	LDH	Hurkmans, D. P., et al. (2021)	238(190-316) [(IU/L)]	92.63 (89.75, 95.6)	110.12 (105.83, 114.58)	IPI	Ahamadi, M., et al. (2017)	IPI-treated: IPI-naïve	107.36 (104.16, 110.6)
						TUMOR TYPE	Hurkmans, D. P., et al. (2021)	Meso: other	58 (32, 84)
Dostarlimab	BW	Melhem, M., et al. (2022)	70 (51- 111) [kg]	87.57 (86.36, 88.77)	121.31 (118.93, 123.8)	SEX	Melhem, M., et al. (2022)	Female: Male	83.8 (79.8, 87.8)

	ALB	Melhem, M., et al. (2022)	3.9 (3- 4.6) [g/dL]	104.1 (101.22, 107.09)	97.51 (95.78, 99.24)
Cemiplimab	BW	Yang, F., et al. (2021)	75 (51- 111) [kg]	68.79 (64.9, 72.92)	146.27 (137.86, 155.19)
	BMI	Yang, F., et al. (2021)	26.5(14.8-56.3) [kg/m^2]	138.57 (125.66, 152.81)	65.57 (57.78, 74.42)

V_C central volume of distribution, P05 5th percentile, P95 95th percentile, 95% CI 95% confidence interval, BW body weight, ALB albumin, LDH lactate dehydrogenase, IPI ipilimumab treatment status: naive or treated, BMI body mass index, [a] reference [31], [b] reference [32]
